# Operational planning steps in smart electric power delivery system

**DOI:** 10.1038/s41598-021-96769-8

**Published:** 2021-08-26

**Authors:** M. Jayachandran, Ch. Rami Reddy, Sanjeevikumar Padmanaban, A. H. Milyani

**Affiliations:** 1 Department of Electrical and Electronics Engineering, Puducherry Technological University, Puducherry, India; 2 Department of Electrical and Electronics Engineering, Malla Reddy Engineering College, Secunderabad, India; 3 CTiF Global Capsule, Department of Business Development and Technology, Aarthus University, Herning, Denmark; 4 Department of Electrical and Computer Engineering, King Abdulaziz University, Jidda, Saudi Arabia

**Keywords:** Electrical and electronic engineering, Energy infrastructure

## Abstract

This paper presents a comprehensive review of advanced technologies with various control approaches in terms of their respective merits and outcomes for power grids. Distributed energy storage control is classified into automatic voltage regulator and load frequency control according to corresponding functionalities. These control strategies maintain a power balance between generation and demand. Besides, three basic electric vehicle charging technologies can be distinguished, i.e. stationary, quasi-dynamic and dynamic control. For realizing charge-sustaining operation at minimum cost quasi-dynamic and dynamic strategies are adopted for in-route charging, while stationary control can only be utilized when the electric vehicle is in stationary mode. Moreover, power system frequency stability and stabilization techniques in non-synchronous generator systems are reviewed in the paper. Specifically, a synchronverter can damp power system oscillations and ensure stability by providing virtual inertia. Furthermore, it is crucial to manage the massive information and ensure its security in the smart grid. Therefore, several attack detection and mitigation schemes against cyber-attacks are further presented to achieve reliable, resilient, and stable operation of the cyber-physical power system. Thus, bidirectional electrical power flows with two-way digital control and communication capabilities have poised the energy producers and utilities to restructure the conventional power system into a robust smart distribution grid. These new functionalities and applications provide a pathway for clean energy technology. Finally, future research trends on smart grids such as IoT-based communication infrastructure, distributed demand-response with artificial intelligence and machine learning solutions, and synchrophasor-based wide-area monitoring protection and control (WAMPC) are examined in the present study.

## Introduction

**Figure 1:**
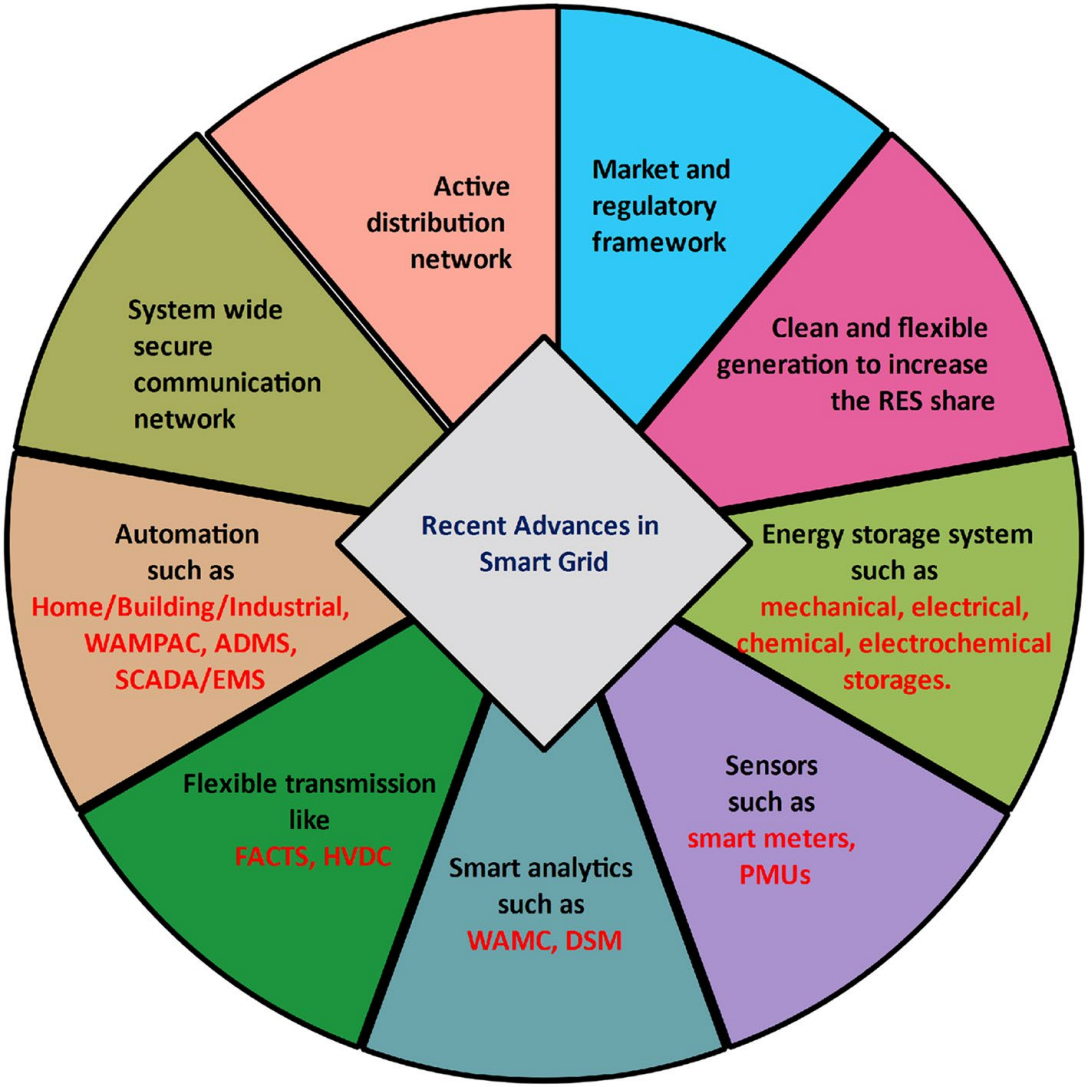
Recent advances in smart grid.

An electricity network that uses digital technology to monitor and manage the energy flows automatically from generating sources to electricity demand is termed as smart grid. This modernized electrical grid employs intelligent monitoring, control, communication, and self-healing technologies to minimize the costs and environmental impacts while maximizing system resilience, security, efficiency, reliability, flexibility, and stability. The smart grid enables active participation by consumers in demand response applications and provides higher power quality. It can accommodate all generations as well as storage systems and operate resiliently against physical and cyber-attacks. Recent advances in the smart grid have facilitated a smart and secure distribution network that requires some features illustrated in Fig. 1^1^.

Energy storage system such as pumped storage hydro (PSH), compressed air energy storage (CAES), flywheels, supercapacitors, superconducting magnetic energy storage (SMES), fuel cell, lead-acid batteries, sodium-based batteries, Li-Ion batteries, flow batteries, and zinc-based batteries utilized in distribution network offers voltage support, peak shaving, power quality improvement, spinning reserve, capacity firming, load leveling and frequency regulation^2^. Compared with the performance, safety and degradation of electrical energy storage systems, Li-ion batteries are the most widely deployed in the power system. Concerning the cost-effective approach to large-scale electric energy storage, smart grid technologies play a vital role in minimizing reliance on energy storage system (ESS) and adjusting the electricity demand. When designing a cost-effective storage system, it is essential to consider all possible factors such as discharge time, storage density, and storage capacity. However, the location of ESS is becoming a common trend in smart grid research. Recent evidence suggests that the energy storage system co-located with photovoltaics (PV) produces the provision of ancillary services, energy shifting, reducing energy curtailment, smoothing generation output, and cost-saving^3,4^.

In the modern age, many advanced technologies have been employed to integrate large-scale Electric Vehicles (EVs) with the power grid. Vehicle-to-Grid (V2G) technology facilitates the interaction between the power grid and EV to ensure the reliability and sustainability of the power grid. Moreover, the V2G concept provides ancillary services such as voltage and frequency regulation and spinning reserve to the power grid. However, the EV charging system design for reliable power supply is crucial for EV demands. Recent evidence suggests that the flexible operation of the EV charger can interact with smart homes, microgrid, and power distribution grid. This mobile energy storage technology with aggregators provides opportunities for the next revolution in the electrical power grid for the benefit of energy consumers and power utilities^5^.

Regarding measurement and sensing technologies, advanced metering infrastructure (AMI) including smart meters with the associated information and communication technology (ICT), power quality management (PQM), outage management system (OMS), peak load management (PLM), distributed generation (DG), Microgrids, Smart Home Solution (SHS), and Smart Analytics (SA) incorporated on existing distribution system evolve the power infrastructure *smarter*. Sensing and measurement technologies enable the transformation of data into information. It evaluates the equipment’s health, grid integrity, and support advanced protective relaying. AMI facilitates monitoring and control through smart meters installed at consumer premises. It supports bidirectional communication between consumer and utility control centers. The advanced functionalities of AMI include accurate load characterization, real-time electricity pricing, and outage detection/restoration. The smart meter records the production and consumption of electricity at regular intervals, whereas the net meter only records the surplus power generation. Smart meters facilitate the record of electricity usage information, remote connect/disconnect switch, and Home Automation Network (HAN) gateway. It also enables Time of Use (TOU) and Real-Time Pricing (RTP) rate metering for Demand Side Management (DSM). The benefits of smart energy infrastructure are improved reliability, supply integration, shorter outages, increased efficiency, consumer cost-saving, and customer satisfaction^6^. Besides, an integrated network monitoring system provides a complete view of the system’s health and faults as well as performance data from different network elements. This monitoring system optimizes the network resources for the network planning process to improve performance and quality of service. Expedited smart grid network diagnostics and decreased network operating costs are other benefits of network monitoring system^7^.

**Figure 2:**
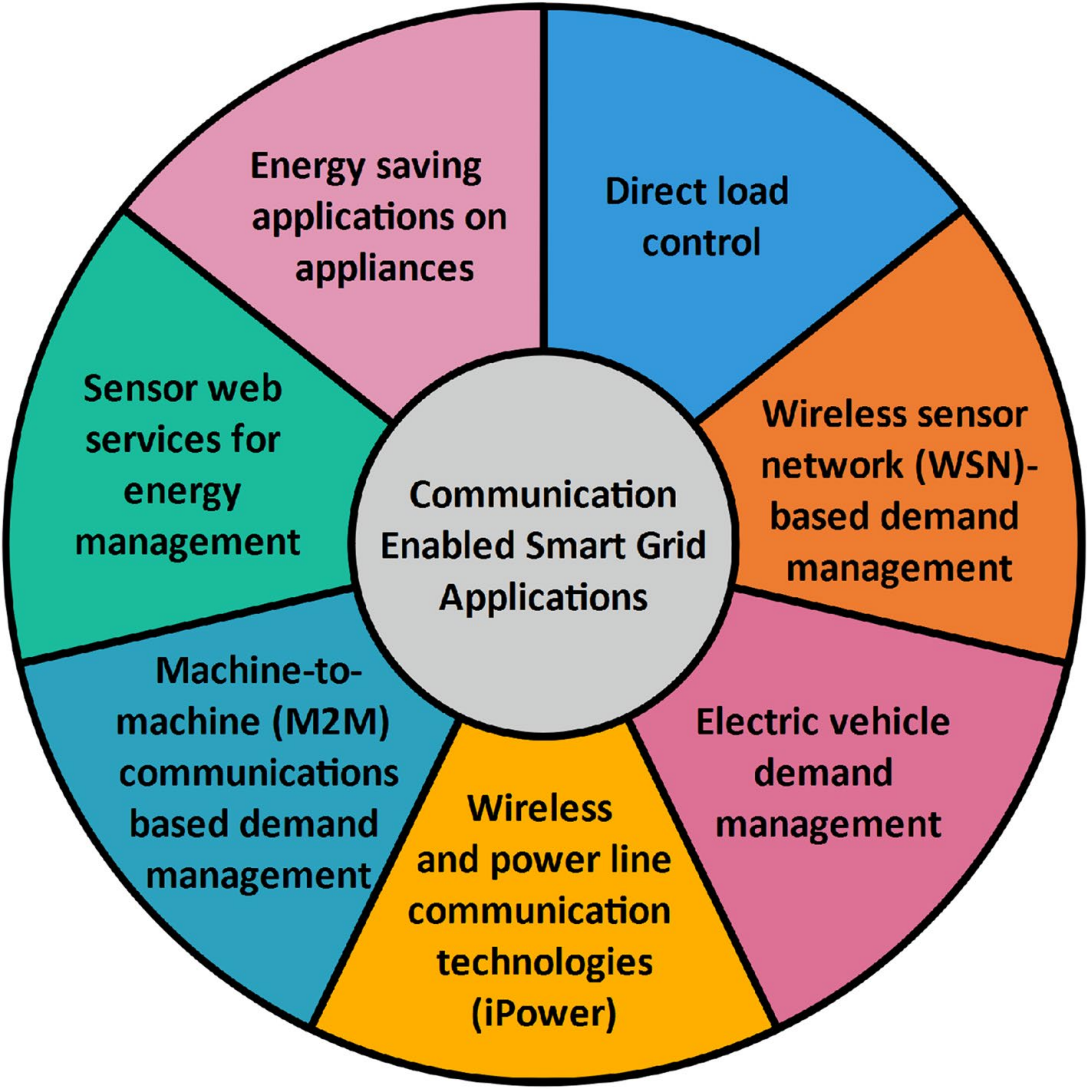
Communication enabled smart grid applications.

As far as communication is concerned, information and communication technologies enable the existing power grid to turn into the smart grid through information and data exchange. The communication-enabled smart grid applications are presented in Fig. 2. Utility commands delivered to the customers through smart meters is known as Direct Load Control (DLC). Sensor web services for energy management enable users to learn the energy consumption of their appliances, load shedding for the utilities, and monitor and control the stored energy as well as energy exported to the grid. For energy saving, the residential gateway can turn-off the appliances based on the threshold when they are in standby mode^8^.

In the case of Demand Response (DR), consumers will be compensated for detaching their load during peak demand using their smart meters and Home Energy Management systems (HEMS). The energy management units and appliances communicate wirelessly over the wireless sensor network (WSN). However, WSN in the smart grid has few challenges such as resource constraints, severe environmental conditions, packet errors, variable link capacity, and reliability as well as latency requirements. Besides, transferring the data and power through the same conductor is known as power line communication. The combination of wireless and power line communication technologies is termed as an iPower. It implements an intelligent and personalized energy conservation system by WSN^9^. Apart from that, Machine-to-machine (M2M) communications based demand management provides load shedding capabilities to utilities during critical peaks and enables remote access to appliance energy consumption^10^. Furthermore, electric vehicle demand management controls its charging and discharging profile based on the solar power generation unit and the demands of smart appliances^11,12^.

Concerning the cybersecurity in power systems, the new communication mechanism should consider security and reliability. Several research works have been published in recent years on unauthorized access, hardware as well as software from attacks, protection of the networks, and rejection of services. Besides, a cybersecurity framework is established for securing Industrial Control Systems (ICS) to support the reliable operation of the grid to ensure secure power delivery. This security process includes identifying infrastructure, assessing threats/vulnerabilities/ risks, implementing security controls, verifying the implementation of security controls, and ensuring compliance to audit^13^. The international security standard for power control systems specifies the security requirement for generation, transmission, distribution, and trading of electric power^14,15^. Besides, the primary goal of the smart grid is to deploy large-scale distributed energy resources (DERs) with power electronic interface, microgrids, and wide-area monitoring, protection, and control (WAMPAC) in a distributed and networked manner. The real-time WAMPAC can facilitate the economic operation of the system, analyze system vulnerabilities, ensure acceptable power quality to consumers, take preventive as well as corrective control actions, and ensure supply-demand balancing^16^.

Some reviews of smart grid advancements have been published in the recent literature^17–19^. Reference^17^ investigates the generation expansion planning on future power systems. Machine learning approach is studied for smart electric power systems^18^. In^19^, a smart grid system has been analyzed with a systemic tool in power grids. However, communication-enabled smart grid implementation issues have not yet been reported in the literature. Therefore, this study focuses on the planning, operation, and control of smart and secure electric power networks.

This review is beneficial for market participants, systems operators, and policymakers. For instance, the present investigation will entice new market participants, enabling a variety of new load management, distributed generation, energy storage, and demand-response options and opportunities. Moreover, the current study enables grid operators to see further into the system and allows them the flexibility to better manage the intermittency of renewables. Besides, such a review facilitates the policymakers to impart clarity to the smart grid and the issues that surround it to make it a reality. The rest of the paper is organized as follows: “Energy storage technologies in smart distribution networks” presents various control strategies of an energy storage system in an electrical power network. “Electric vehicle charging station (EVCS) and its impact on smart distribution grid” analyzes EV charging infrastructure and its impact on the distribution system. “Coordinated control, protection and stability of modern power systems” explores the planning, operation, and control of modern power systems with stability enhancement. “Microgrid resiliency and cyber-physical power system” investigates the overall structure of cyber-attacks on smart-grid control systems to improve cyber resilience in the power system. Then, the future trends on the modernization of the electric power system are summarized in “Discussion on future trends”. Finally, this paper concludes in “Conclusion”.

## Energy storage technologies in smart distribution networks

Over the past decade, distribution networks (DNs) have operated with conventional control strategies. The integration of MW scale solar energy in distribution power grids, using an energy storage system, will transform a weak distribution network into a smart distribution grid. In this regard, more research is required for voltage control. To enhance the flexibility of distributed generations and provide ancillary services, active distribution networks are also necessary for transmission network support. Smart grid technologies are evolved for voltage control in DNs to achieve the best services. However, a reliable and robust system operation will be the main research challenges. Some studies suggest that optimization-based techniques are one of the most attractive control schemes to obtain loss minimization and voltage regulation^20^. Renewable power generation and load uncertainties, and network disturbance are main concern that affects the network voltages. Therefore, further research is essential to regulate network voltages using smart grid technologies in DNs with real-time measurements. Managing the reactive power exchange between transmission and distribution networks by utilizing DG units is a potential concern for future research. Few studies have analyzed the causes of DGs on maximizing the reactive power output. However, these methods fail to meet the requirement at point of common coupling (PCC) when more than one network is integrated. Recently, there has been extensive research on transmission network support by DNs, either maintaining the voltage at PCC or reactive power exchange between transmission and distribution network within a specified range^21,22^. Future studies should focus on a centralized real-time short-term voltage control strategy for DNs. This work can be extended to investigate the interaction between DNs with high voltage network during transient and steady-state conditions. The development of robust, reliable, and cost-effective technology to each energy source is yet another prominent research topic.

### Planning and operation of energy storage in DSO grid

The traditional Distribution Network Service Provider (DNSP) is required to evolve into a flexible Distribution System Operator (DSO) for emerging smart grids in which generators, consumers, and network elements have actively managed to accomplish technical, environmental, and economic objectives. In general, there are certain technologies that may not be possible for network operators to manage storage, distributed production, etc. However, the Hungarian Energy and Public Utility Regulatory Authority had granted a possibility for distribution system operators (DSO) to install, operate, and control the electric energy storage systems to optimize power distribution using the least cost principle for PV based power networks^23^. For a better operation of the distribution system, information exchange is essential between the DSO and the customers. Binary Controlled Step Position Information (BSC) controller is an essential component of the battery management system (BMS). The control objectives of BSC control are to operate the energy storage inverter (ESI) within the specified limits, control ancillary equipment, and communicate with top-level management. The establishment between the control center and the substations can transform the passive low voltage distribution network into an active smart distribution grid.

In reviewing the literature, the voltage control module utilizes either hysteresis-based or internal model control (IMC). In hysteresis-based voltage control, as proportional control, does not use a network model, and the control parameters must be chosen very carefully to reduce oscillations. IMC-based voltage control, as an integral control, uses a network model that is characterized by the droop parameter. Droop values can be ascertained by experimental methods. However, small-signal modeling and stability issues of the complex network is a very challenging research task. The connection point voltage varies due to unknown disturbances. To establish a stable PCC voltage, the controller determines the required active power using desired and measured voltages. The power signal then passes through the power controller. This power control module protects the battery energy storage system (BESS) and controls the ESI. To summarize the network level control, the IMC based control significantly reduces voltage fluctuations of the affected feeders^24^. Although BESS fulfilled the requirements, further modifications are needed in the control concept to obtain the best results.

### Distributed energy storage systems in wildfire events

Recently, wildfire events increase the risk of electricity grid damage resulting in blackouts.

Exploring solutions for providing continuous power supply to consumers under wildfires is a very active field of research. Incorporation of distributed energy storage system (DESS) into the smart grid can effectively reduce wildfire impacts leads to improving grid resilience and reliability.

#### Before wildfire events

For minimizing the effect of primary grid failure, grid integration of DESS can contribute preventive response such as (1) forming decentralized energy systems, (2) isolate the system from the grid, (3) Increases the robustness of the system while providing virtual inertia, and (4) Maintain the stability of the system by mitigating the disturbances.

#### During wildfire events

DESS can facilitate proactive and corrective actions such as (1) system islanding, (2) network reconfiguration, (3) optimal dispatching, and (4) Optimal load shedding. According to the IEEE 1547 standard, the islanding condition should be detected in 2 s.

#### After wildfire events

DESS increases the system flexibility and perform an optimal power system restoration process. It includes (1) maintaining supply-demand balance, (2) making renewable resources dispatchable, (3) increasing the amount of served load, (4) providing reserve capacity, and (5) optimize the distribution of power flows^25^.

Along with this growth in the smart operation of DESS in reducing wildfires impacts on smart grids, however, there is increasing concern over energy storage technologies. The flammability and explosive characteristics of MW battery storage is a research concern regarding the deployment of battery storage technologies. Of particular concern are fire and explosion hazards in fuel cell technology. Lack of large scale energy storage capacity in energy storage technologies is another potential concern.

### Battery energy storage system in damping control

Maintaining the power system frequency within a specified range is a primary objective of power system planning and operation. Several studies have reported that increasing DER in power distribution networks causes insufficient system inertia, which leads to grid instability^26^. Alternatively, some BESS have designed and operated using advanced control concepts to ensure the output power is proportional to system frequency. BESS with appropriate grid-supportive inverters evolved nowadays for enhancing recovery of power system frequency following a disturbance and thus increases grid stability. Most of the existing methods using DER only consider power system frequency recovery. However, Inertial-support using ESS is not yet established for industrial applications. Besides, the active frequency damping control concept using BESS was proposed recently. The control inputs such as off-nominal frequency, power angle of inverter interfaced renewable generator, and rotor angle of the synchronous generator are applied to BESS control. This charging and discharging control strategy modulates BESS power output in coordinated with an inverter-based generator and rotating generator to minimize off-nominal frequency deviations within the acceptable range. Thus, the overall system is positively damped and improves grid stability and reliability^27,28^. The control input measurements of damping control require a communication channel for time synchronization and high resolution. The merits and outcomes of the various control methods for distributed energy storage architecture in smart grids are summarized in Table 1.

**Table 1:** Smart grid and energy storage system.

Methodology	Control method	Merits	Outcomes
Active distribution network	• Optimization-based voltage control methods	• Voltage correction	• Reducing voltage fluctuation and Maintaining voltage at PCC in distributed network caused by large penetration of PV
	• Real-time short-term voltage control scheme	• Loss minimization	• Ability to support transmission network by improving power factor at PCC
		• Reactive power exchange between transmission and distribution network	
Battery energy storage system in DSO grid	• Hysteresis-based voltage control	• Control the voltage on long radial overhead lines	• Successfully control the voltage of the affected feeders
	• Internal model control-based voltage control	• Control the inverter within the limits set by the battery management system	
Distributed energy storage system in Wildfire	• DESS management system using Smart control mechanism	• Increase grid resiliency	• Improving smart grid reliability and resilience
		• Increase the system flexibility	• Providing continuous and reliable power supply for customers
		• Maintaining supply-demand balance	• Increasing the reliability of power supply in weak distribution system
		• Optimize the distribution of power flows	
Active damping with Battery energy storage system	• BESS active frequency damping control	• Reducing power system frequency deviations	• Reducing frequency fluctuation at PCC in distributed network caused by dynamic loads
		• Providing grid stabilizing inertial-support	• Improving smart grid stability and reliability
			• To accommodate more renewables into power systems before hitting stability limits

## Electric vehicle charging station (EVCS) and its impact on smart distribution grid

As a consequence of the evolution toward smart grids and the expansion in electric transportation, the deployment of electric vehicle charging stations for electric vehicles becomes feasible. As a backbone of EV charging infrastructure, AC level 2 chargers or dc fast chargers play a vital role in the efficient and reliable operation of the overall system. Besides, different types of ESS can be employed in EV charging stations, such as a battery, flywheel, and hybrid energy storage systems. The impact of these storage systems on EV chargers is examined^29^. For providing faster charging to the EV battery, a supercapacitor is interfaced to the system. Moreover, the benefits of installing the EV charger on a distributed grid and its techno-economic effect is studied^30^.

### Renewables for clean energy transportation

When consumer electricity demand is low, the surplus renewable generation can lead to negative pricing frequency at wholesale energy markets. Due to this excess generation, increased penetration of renewables may cause higher renewable generation curtailment. Instead of curtailing that surplus energy, it can store all renewable energy in the form of hydrogen for transportation needs using a chemical process called electrolysis. The negative pricing as a result of more renewable energy on the grid provides cleaner energy-transportation applications such as Fuel cell vehicles (FCV). Therefore, hydrogen storage technology for FCV research in the auto industry should further focus on the FCV range, adaptability, and refueling time of fuel cells^31–34^.

### Planning of in-motion electric vehicle charging system

Concerning the power system, EV aggregator, as virtual energy storage, can provide ancillary services in the electricity market with appropriate energy policy and tariffs. The growing EV market has faced critical challenges of long refueling time and limited driving range for EVs. To address these concerns, install a large on-board battery for maintaining mileage and stationary charging capability. The stationary charger can classify in terms of the medium of conduction into inductive (or) capacitive wireless power transfer, and AC (or) DC conductive charger. However, large on-board batteries increase vehicle cost, size, and energy consumption. It also requires fast charger^35–37^.

**Figure 3:**
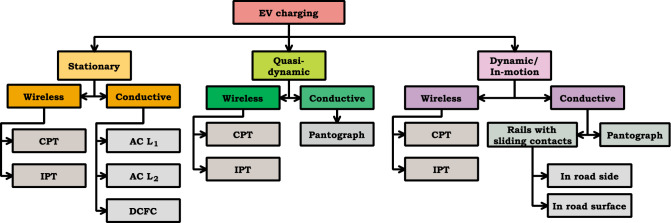
Various charging technologies for EVs.

For realizing charge-sustaining operation at minimum cost, dynamic and quasi-dynamic technologies are widely used for in-route charging, such that EV can charge while movement and transit stop as indicated in Fig. 3. These approaches are developed to extend the driving range and permit the use of a small on-board battery. Quasi-dynamic charging can evolve for secondary roadways with low-speed driving areas as well as transit stops. On the other hand, with continuous high-speed driving, in-motion charging shows a perfect fit for freeways. Both technologies can operate in conductive (or) wireless mode. In particular, proper planning of a dynamic wireless charging system for EVs on highways can realize charge sustaining operation^38,39^.

### Mix energy source EV fast charging station

Two or more devices capable of working together in several distributed applications are represented as Interoperability. Although the dynamic charging system is interoperable, there is a tradeoff between system power and road coverage. A high power system can reduce road coverage, but it requires powerful components which is costly. Several studies have reported various EV system architectures in the power distribution grid. However, research has yet to be systematically investigated. In the case of grid-connected operation, charging of batteries is preferred during off-peak hours. Therefore, grid-connected EV fast charging with auxiliary supply from PV and ESS has been developed in recent years. However, this system requires a high power charging module, which is expensive. For examining this issue, modular multi-port power electronic transformer cascaded H-bridge concept has recently been proposed to achieve multiple and simultaneous EVs charging with low power components in the EV charging station. An advanced control scheme needs to be developed for further work.

### Electric vehicle charging management at EVCS

**Figure 4:**
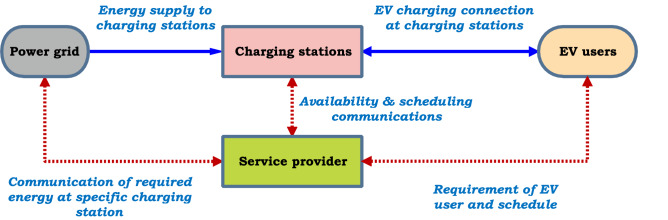
Energy flow and communication in EVCS.

To combat range anxiety in electric transportation, the placement of EV charging stations into existing transport and power networks is one of the principal challenges for enhancing the potential contribution of EV to the power grid. Recent evidence suggests that optimal sitting and sizing of EVCS is determined by an optimization algorithm using the driving pattern of area^40,41^. EV demand and population density can play a vital role in both power and transportation networks^42^. The EVs charging management at the EVCS includes the grid, the service provider (SP), and the EV driver. The scheduling of energy flow and communication among these entities is presented in Fig. 4. Multiple charging locations provide access to SP for managing the EVs recharge to minimize the burden of the grid and maximizing the profit.

Concerning densely populated areas, a larger number of EVs are requesting to recharge the batteries simultaneously at the same location. To resolving this problem, researchers are presented a battery swapping station (BSS) for exchanging a depleted battery with a charged battery with less time^43^. As compared with EVCS, BSS takes less time and offers more convenience and flexibility. Implementation of an energy management system (EMS) for large-scale grid-connected EVs is another potential concern in the V2G application. Besides, the security and privacy challenges in the large scale V2G network and its impact on grid resilience need to be addressed for further studies. The merits and outcomes of the various control methods for mixed energy source EV charging system design on smart distribution grids are summarized in Table 2.

**Table 2:** EV charger and charging infrastructure in smart grids.

Methodology	Control method	Merits	Outcomes
Hydrogen storage for fuel cell Vehicles	• Electrolysis	• Lower solar energy curtailment and store the energy in hydrogen for transportation	• Increased renewable penetration and negative pricing provides clean energy transportation
In-motion charging technologies for electric vehicles	• Dynamic charging	• Solving charge-sustaining model for EV on highways	• Planning of in-motion EV charging for realizing charge sustaining operation at minimum infrastructure cost
Electric vehicle charging management	• EV charging station	• Depleted battery is either charged in EVCS with less charging time or exchanged with a charged battery in BSS	• Appropriate EVCS to be installed into the existing power network
	• Battery swaping station	• BSS offers more flexibility and convenience	• Manage the charging of the EVs at EVCS in densely populated areas
Grid connected EV fast charging station with PV and ESS	• Modular multi-port power transformer cascaded H-bridge concept	• Possible to charge multiple and simultaneous EVs	• Planning of EVCS to maximize the contribution of EV to the power distribution grid

## Coordinated control, protection and stability of modern power systems

The emerging smart grid technologies like volt/var management system (VVM), power quality analyzer (PQA), supervisory control and data acquisition (SCADA), geographic information system (GIS), distribution automation (DA), and AMI are integrated into modern power systems to monitor and analyze power quality issues^44,45^. Voltage/VAR control, harmonics control, and load balancing are performed through PQM to reduce losses, minimize equipment failures, improve customer satisfaction, and facilitate reliable as well as efficient power system operations. To manage distribution transformers, HT/LT feeders, the OMS collects outage information from customers and coordinates with the operator for corrective actions through remote control. Thus, OMS improves system availability and reliability, and enables customer satisfaction. Moreover, for balancing the demand and supply at the peak instant to avoid load shedding, PLM collects the information about power availability and load demand from SCADA/EMS and suggests the corrective actions to the system integrator in association with utility personal. This approach manages peak load by either ToU pricing or load curtailment through AMI. Furthermore, the flexibility of interaction with customers, energy trading/transaction management, and demand/loss management can be achieved through distributed generation technologies, systems, and solutions^46^. Finally, A micro-grid is an integrated DGs with a communication system and loads, which operates in isolated mode during normal operations or grid-connected mode in case of emergencies. This isolation capability from a larger network provides highly reliable electric power to its consumers. Expanding DER capacity improves economic feasibility. The essential feature in DER interconnection is capable of detecting operation under an islanded condition. IEEE standard 1547.6 represents the practice for interconnecting distributed resources with electric power system distribution secondary networks^47^.

### Frequency stability and control of modern power system

Frequency stability and control is a challenging problem in the design and operation of interconnected power networks. Maintaining power system frequency within the specified operating range in a power system is referred to as frequency stability. The insufficient active power generation under heavily loaded power system affects frequency stability. Hence, the imbalance between generation and load with poor coordination of control results in frequency instability.

**Figure 5:**
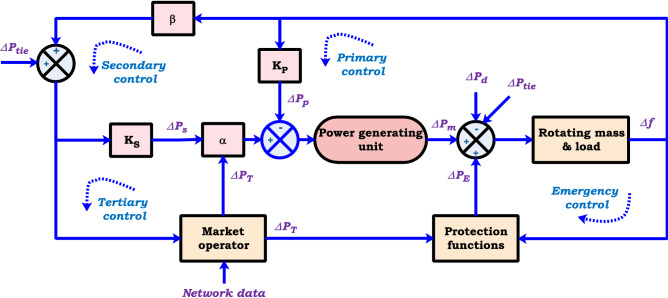
The conceptual block diagram of frequency response.

Conventional frequency response model in particular all described frequency control loops is performed to maintain power system frequency stability as conceptually shown in Fig. 5.

Primary control, as proportional control, can attenuate small frequency deviations to keep the system frequency at a fixed value. Secondary control, as a load frequency control (LFC)/automatic generation control (AGC), is to eliminate large frequency deviations and restore the system frequency to its nominal value. Tertiary control can decrease rapid frequency changes following a significant fault, and hence the power ratio between the supply and demand is balanced. In addition to hierarchical control, the emergency control and protection function minimize the risk of cascade outages, load/network separation events, and additional generation events using standby supplies^48–50^.

### Virtual inertia control to improve frequency stability of modern networks

**Figure 6:**
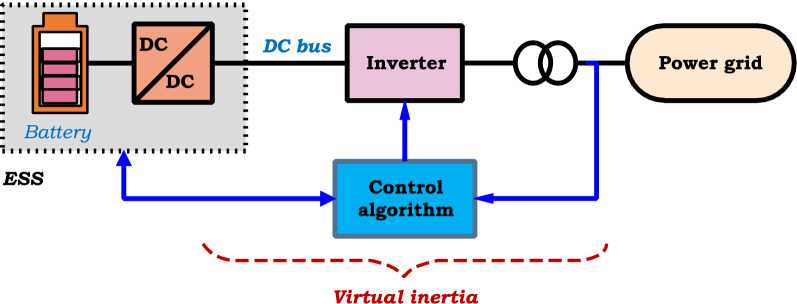
Schematic diagram of grid-connected virtual inertia system.

Increasing renewable energy penetration through voltage source converters (VSCs) into modern power systems with a reduced number of synchronous machines leads to a reduction of system inertia resulting in frequency instability. Inertia emulation and active power injection from controlled power sources provide opportunities to handle frequency control in modern power grids. A virtual inertia (VI) system has been established in recent years using ESS and VSC to achieve a proper inertial response, as depicted in Fig. 6 with an appropriate control structure. The control method should be able to emulate the required inertia and behave the system as a synchronous generator. Thus, VI ensures the short-term frequency stability of the system^51^.

**Figure 7:**
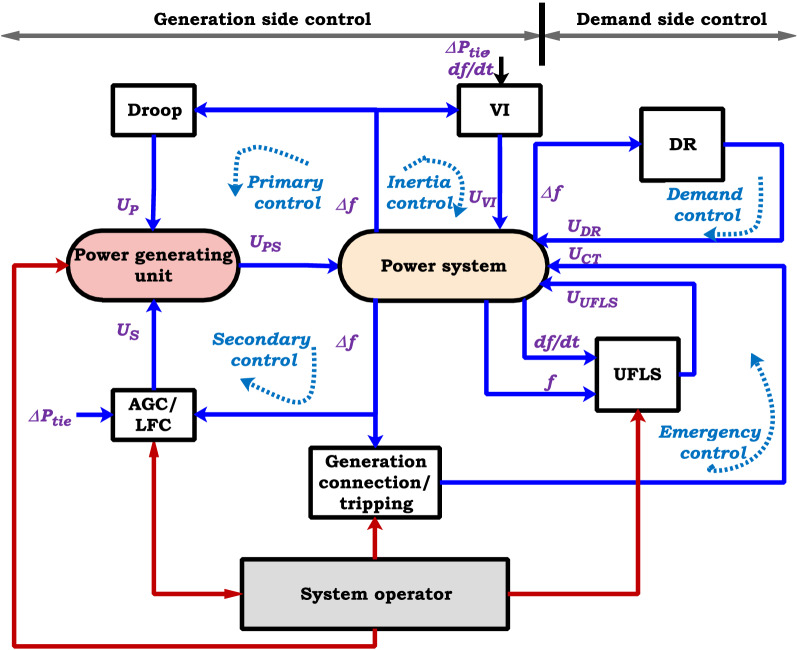
Updated frequency control scheme in modern power system.

The updated frequency control loops in the modern power system with new control possibilities are presented in Fig. 7. By injecting active power into the distribution system after severe generation/load disturbance, DG/MG can support the conventional synchronous generator during the activation of the prime reserve. ESS can enhance the power system resiliency and dynamics, which rely on variable generating units. In this way, ESS would be appropriately controlled to emulate virtual inertia^52^.

### Stability enhancement ancillary service in power systems

The inverter-interfaced distributed renewable energy sources (DRESs) are highly controllable that provide ancillary services such as active power ramp rate control, frequency sensitive mode operation, inertial response, voltage regulation, harmonic mitigation, and fault clearing. A conventional VSC is replaced with a synchronverter to overcome the deficit of inertia in the power system using proper control strategy. A Synchronverter, a combination of synchronous generator and inverter, is used to produce both fixed and variable amounts of inertia as required to improve the system stability^53–55^.

Demand response, as controllable loads, provides ancillary services for frequency regulation. The centralized switching-based control with the recent advances in control, communication, and computing technologies enables the loads to respond faster to system disturbances^56,57^.

**Figure 8:**
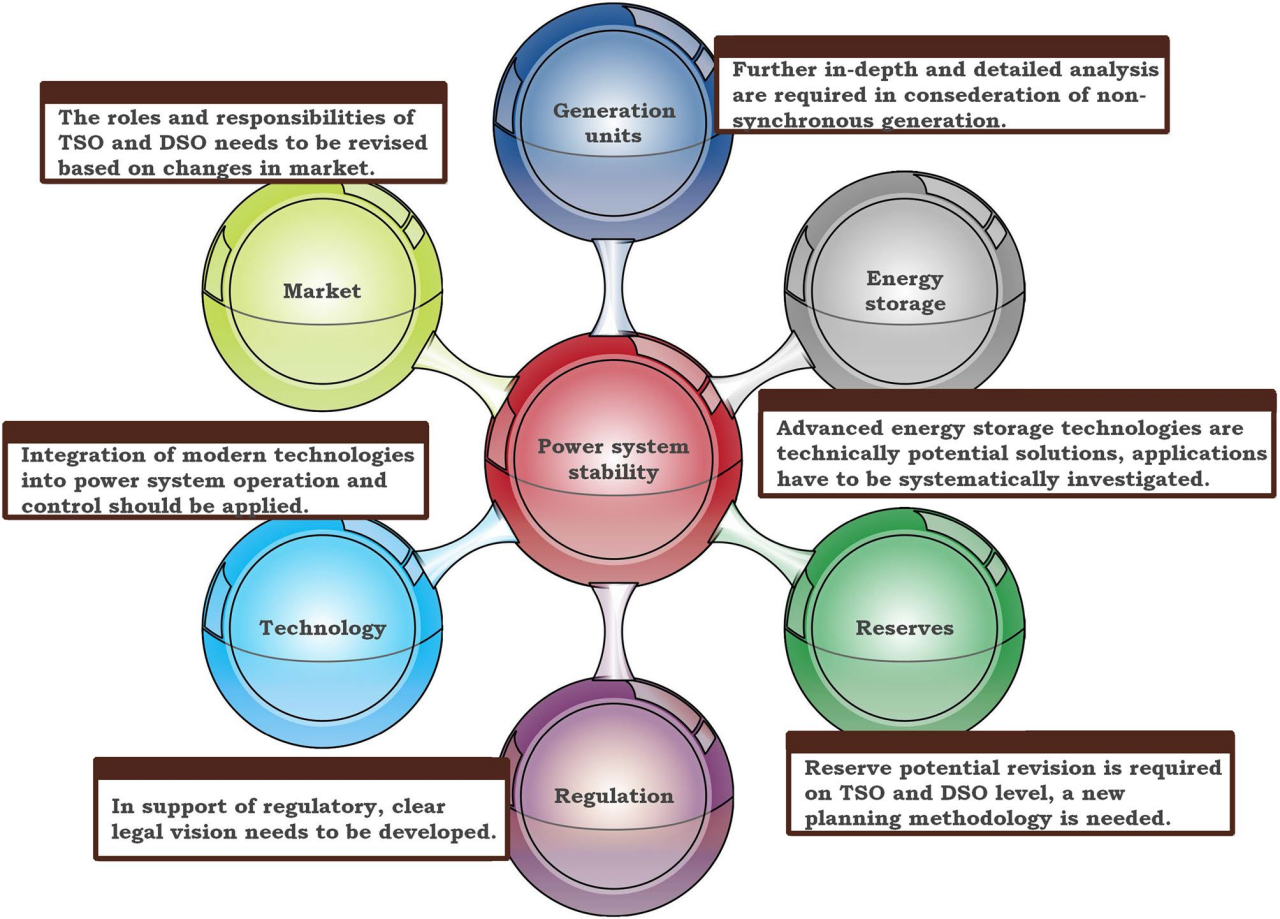
Various aspects regarding power system stability in non-synchronous generator systems.

In summary, the integration of DGs, controllable loads, virtual inertia, and wide-area measurement systems provide new frequency control opportunities in the smart grid for future research. Moreover, for ensuring system stability, advancement in control concept within this field enables smart grid solutions. Further study should focus on the integration of non-synchronous generators into the reserve provision through virtual inertia, virtual synchronous machine, and fast frequency response to gain practical experience. The summary of various aspects regarding power system stability is presented in Fig. 8.

### Energy management in smart grid

In general, renewable energy generation does not coincide with the load profile. Therefore, load management and control are required for demand response and tariffs. The objective of demand response is to minimize both electricity cost and aggregator’s power consumption. For maximizing the supply-demand balance efficiently, EMS is installed to increase the reliability and efficiency of power and distribution systems. The electricity supplier can use the EMS to control its generation units in an efcient way. For instance, to meet a certain power demand of the consumers, using energy management, the electric utility can turn on some generators, which may have the least operation cost, while the generators with high operation cost are left to supply extra load demand in specic peak periods. In this way, the electric utility is trying to minimize the operation cost of its generation units. With increasing penetration level of RES and minimizing energy loss in the network, the system operator uses EMS to regulate power flow. To lower the electricity cost and schedule the load demand, energy consumers can use EMS to reduce the peak load during peak hours. Besides, DSM strategies such as utility-driven, consumer-driven, and multi-objective strategies serve in reducing consumption during peak demand with industrial customers. Providing clean energy to customers during peak demand is another promising solution to reduce ratepayer costs^58–61^.

Most existing studies in the field of energy management have only focused on developing models and sophisticated algorithms. For a better future, integrating information and communication technologies to advanced control systems such as PLC, SCADA, EMS, BMS, and automation systems with a smart algorithm, the conventional grid becomes a smarter one to manage energy on the grid in an efficient way. The fundamental functions of the distribution management system (DMS) are state estimation, network connectivity analysis, load flow analysis, Volt-Var control, intelligent load shedding and restoration, fault management and system restoration, load balancing through feeder reconfiguration, and forecasting in distribution networks. The advanced distribution management system (ADMS) approach has received considerable scholarly attention in recent years, and the functions are training, planning, optimize, operating, analyzing, and monitoring the distribution network. ADMS includes SCADA, DMS, OMS, and DSM to analyze and manage DER. It can support closed-loop control and automated switching for self-healing. As grid parameters changes, ADMS executes the grid optimization program for monitoring and adapting volt/VAR settings automatically. Moreover, it offers many optimizations and grid improvement functions for demand and efficiency management. The ADMS concept will provide a platform in the future smart grid to coordinate DMS and micro-grid EMS for better energy management and stability^62–64^.

### Data analytics in demand response applications

The demand and supply gap is a critical issue in the smart grid. To date, numerous studies have reported several solutions for demand response applications in the literature. However, data analytics is one of the most successful techniques for analyzing the gathered data to regulate the power flow in the smart grid environment. In a smart grid, entities from generation, transmission, and consumption generate the energy-related information. These entities are connected to the internet for transferring data, thereby forming massive internet of energy. Such data can be analyzed for managing conventional or non-conventional sources efficiently to the end-users. Thus, data analytics play a pivotal role in reducing the demand and supply gap and regulate power flow from generation to end-users^65^.

Data analytics can be categorized into descriptive, diagnostic, predictive, and prescriptive analytics. With the knowledge of demand and supply, these analytical methods perform demand response management, which helps to optimize the electric consumption of consumers for minimizing the burden on the smart grid. The practical advantage of using data analytics for demand response management includes better capital expenditure, increased customer satisfaction, enhanced efficiency, and reliability of the smart grid. However, data sharing, security, and privacy are research challenges for future research work. Also, real-time AMI data integration into the power grid is another potential concern for demand response applications. Variability of response and infrastructure requirements are needed to be considered further for managing and analyzing the demand response^66^.

### Wide band gap power semiconductor devices

Modern power converters require efficient power semiconductor devices for processing and delivering of clean electrical energy to consumers. Therefore, new power electronic devices based on wide bandgap semiconductor devices such as silicon carbide (SiC) and gallium nitride (GaN) have developed and commercialized in recent years^67^. Compared with traditional silicon devices, a wide-bandgap GaN device has the advantages of high electron mobility, large bandgap, and low dielectric constant. Thus, these devices are more suitable for fast-switching and high-power-density power electronics converters in renewable energy system smart grid applications owing to its high voltage rating and high power handling capability. Low contact resistance, small source-drain separation, and sharp edge acuity are the main factors for high-power and high-frequency applications. In high electron mobility transistor (HEMT), the metal contacts are fabricated on the GaN material with aluminum gallium nitride (AlGaN) barrier layer to develop AlGaN/GaN power transistor for smart grid applications^68^.

For high power applications, SiC power devices are preferred in a three-phase system, which typically requires more than 1200 V, because of its high breakdown voltage, high current density, and high thermal conductivity. In medium and low power applications, GaN-based power devices are utilized. Due to the high critical electric field and high electron mobility, these devices are the dominant choice for high-frequency applications. Moreover, 600 V GaN/SiC power semiconductor devices can be used in single-phase PV inverters, EV on-board charger, and wireless power transformer chargers^69^. The merits and outcomes of the various co-ordinated control methods for stability of smart distribution grids are summarized in Table 3.

**Table 3:** Smart grid stability and control.

Methodology	Control method	Merits	Outcomes
Monitoring communication and computing	VVM, AMI, GIS, SCADA, DA and PQA	• VVM can regulate distribution system voltage profile and minimize reactive power flow	• Planning and deployment of monitoring system to monitor and analyze the power quality problems
		• FILR capability	• Provide clean power to end-user equipment with minimal disturbances
		• PQA can Investigate disturbances in power system	
Integration of non-synchronous generators into reserve provision	• Virtual synchronous generator	• Artificially add inertia through converter controls	• Synchronism is ensured in large scale interconnected power systems
	• Fast frequency response control	• Frequency gradient has been reduced in small island power system.	• Ensure system stability
Virtual inertia	• Voltage source converter control Synchro-converter	• Increasing the inertia of the system	• Enhancing ancillary services by virtual inertia emulation to regulate frequency in active distributed network
		• Enhance frequency stability profile of the system	• Maintain power system frequency stability by providing virtual inertia and Ensure the long-term frequency stability of the system
		• Damp the oscillations in the power system	
Data analytics	• Descriptive, diagnostic, predictive and prescriptive	• Reducing demand supply gap	• Finding the optimal solutions for managing the load of end-users in smart grid
		• Regulate power flow in generation, distribution and consumption.	
Wide band gap power semiconductor devices	• AlGaN/GaN-based HEMT devices	• Improve power density and power efficiency	• HEMT based Technology meeting the needs for power distribution grid and end-use utilization

## Microgrid resiliency and cyber-physical power system

Considering physical, application, information, and infrastructure domains, a complete a holistic cybersecurity framework encompassing attack deterrence, attribution (forensics), prevention, detection, mitigation, and resilience for the smart grid are essential for reliable power grid operations. Artificial intelligence and machine learning-based real-time intrusion prevention/detection systems are some of the most successful methods used for malware detection, classification, and mitigation in smart grids. Network segmentation is another possible solution to improve the efficiency, sustainability, cyber-security, resilience, and reliability of electricity services in the smart grid. Besides, controlled wireless propagation and authentication techniques have been proposed for the smart grid without affecting any proper operations. These solutions build a smart, scalable, secure, resilient, and adaptive cyber-physical power system^70^.

### Resilient energy distribution system

A microgrid is a small-scale and self-adequate power distribution system. It consists of RES, ESS, and loads with coordinated control strategies. loads within a microgrid can be supported by its local distributed generators continuously, which enables the MG to be disconnected from its upstream macro-grids during extreme events or contingencies. This salient feature maximizes grid resiliency. A resilient power system should be capable of withstanding, anticipating, and responding to unprecedented events^71–76^.

#### Enhancing power system resiliency

The Telecom industry, hospitals, defense sector, and water utility require a highly resilient power system since they are directly related to the country’s economy. In a stand-alone microgrid, diesel generators and micro-turbines have been used for backup supply at load centers. However, fuel and operational costs are expensive. The potential solution is to form sustainable microgrids, which include RES and ESS. The sustainable microgrids with an advanced control strategy can enhance power system resiliency^71^. In grid-connected sustainable microgrid can generate revenue through efficient energy management and demand response in smart grid technologies. Therefore, an increase in resiliency playoff-high investment and further study should ascertain the feasible solution between resiliency and cost.

#### Enhancing grid resiliency

Multiple microgrids are connected to form a networked microgrid for enhancing resiliency benefits. A distributed system with a networked microgrid can exchange power with each other to achieve economic and efficient operations for improving resiliency benefits^72^. Considering distributed renewable resource uncertainties, load fluctuations, and interdependency of microgrids, the real-time challenge in existing networked microgrids is to ensure supply and demand balance within the individual or networked microgrids^73^. A recent study reveals that the problem can be formulated as a centralized/decentralized optimization model using game theory or a reinforcement learning-based approach ensuring both system resiliency and economic efficiency^74,75^. Conversely, according to IEEE standard 1547.4, sectoring distributed grids into several self-adequate microgrids through reconfiguration can improve system reliability. However, during sectionalization, voltage/frequency stability within newly formulated dynamic networked microgrids is another challenging task. For smooth sectionalization of distribution grids into autonomous microgrids through intelligent system reconfiguration during extreme weather events, the fault-tolerant control is more recently developed for self-healing to provide continuous power supply to the maximum number of consumers^76^.

Although self-adequate microgrids offer several benefits to the energy distribution sector, it requires an additional investment cost in communication infrastructure. Therefore, further research should focus on the economic feasibility of networked microgrids for the future resilient energy distribution system.

### Cyber-security in smart power grid

Information communication technologies can enhance the quality, efficiency, and reliability of smart grids. However, these technologies cause unwanted threats in the system. Cyber-attack is one of the significant attacks in the smart grid control system (SGCS). By injecting false data on/denying actual data from single/multiple sensors, communication links, and actuators in the control system to disorient the system objective by a third party in an unauthorized manner is known as a Cyber Attack. It can either be simple to detect or smart/coordinated, which cannot be identified by the existing fault data detection schemes. A cyber attack is developing new ways to detect and prevent such cyber threats in SGs will enhance the power system resilience.

**Figure 9:**
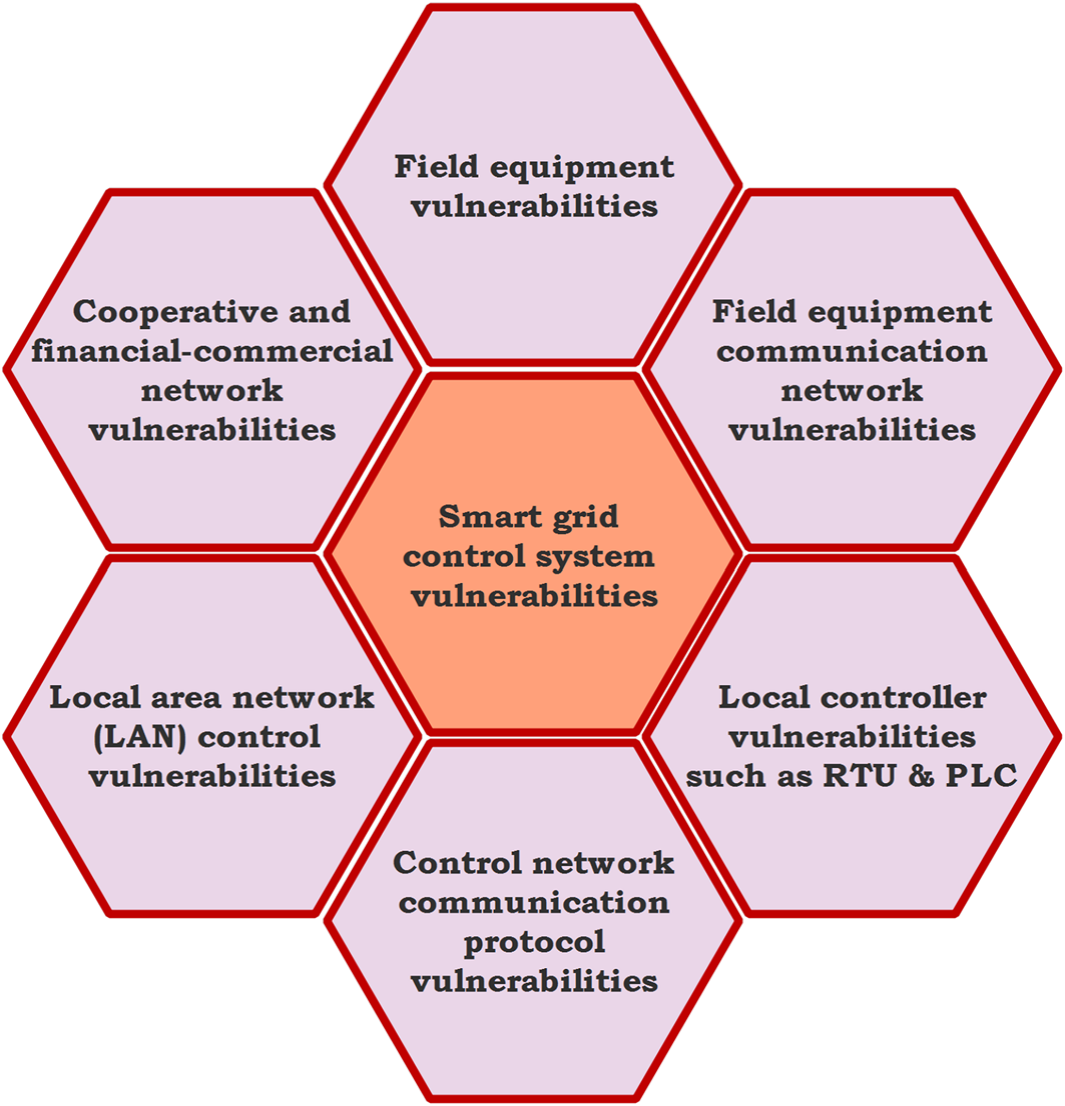
Control system vulnerabilities in smart grid.

SGCS consists of field equipment communication networks such as sensors and actuators, a local controller such as remote terminal units (RTUs) and programmable logic controllers (PLCs), control networks communication protocols like computers and switches, local area network (LAN) control, and co-operative commercial networks^77^. The vulnerabilities of these components used in SGCS is depicted in Fig. 9. The control and protection system used in SGs utilizes the same software and network platforms with the same standards. As a consequence, unauthorized persons may access, attack, and damage an intelligent control system. In order to secure SGCS, IT-based security strategies are required to identify and analyze the communication vulnerabilities of communication protocols and equipments^78–89^.

#### Communication vulnerabilities

Industrial intelligent control system communication protocols are designed for enhancing efficiency and reliability operations in SGCS. These protocols may support economic and operational requirements. Due to the lack of security features such as authentication and cryptography, they are vulnerable and exposed to many attacks^78^. A variety of industrial protocols, their vulnerabilities, and IT-based security solutions have been addressed in literature^79^. One way to increase the security of communication protocols is the mod-bus protocol that uses a model-based intrusion detection system for monitoring and detecting the Modbus TCP protocol attack. Moreover, firewalls are employed for security to prevent unauthorized access to the prevented system^80^. For ensuring data confidentiality and integrity, various encryption methods have been presented in Ref.^81^.

#### Control equipment vulnerabilities

One of the most critical software vulnerabilities in control equipment for the industrial control system is the Stuxnet attack, which targets the SCADA system via vulnerabilities^82,83^. To counteract this attack, the security expert proposes security measures for a power grid state estimator for protecting a limited number of measurements and their output to prevent the data injection attack. For maximizing the security against injection attacks, an optimal defense strategy has been developed to minimize the amount of damage and provide protection for a limited number of devices^84^.

#### Attack on power grid control system

**Figure 10:**
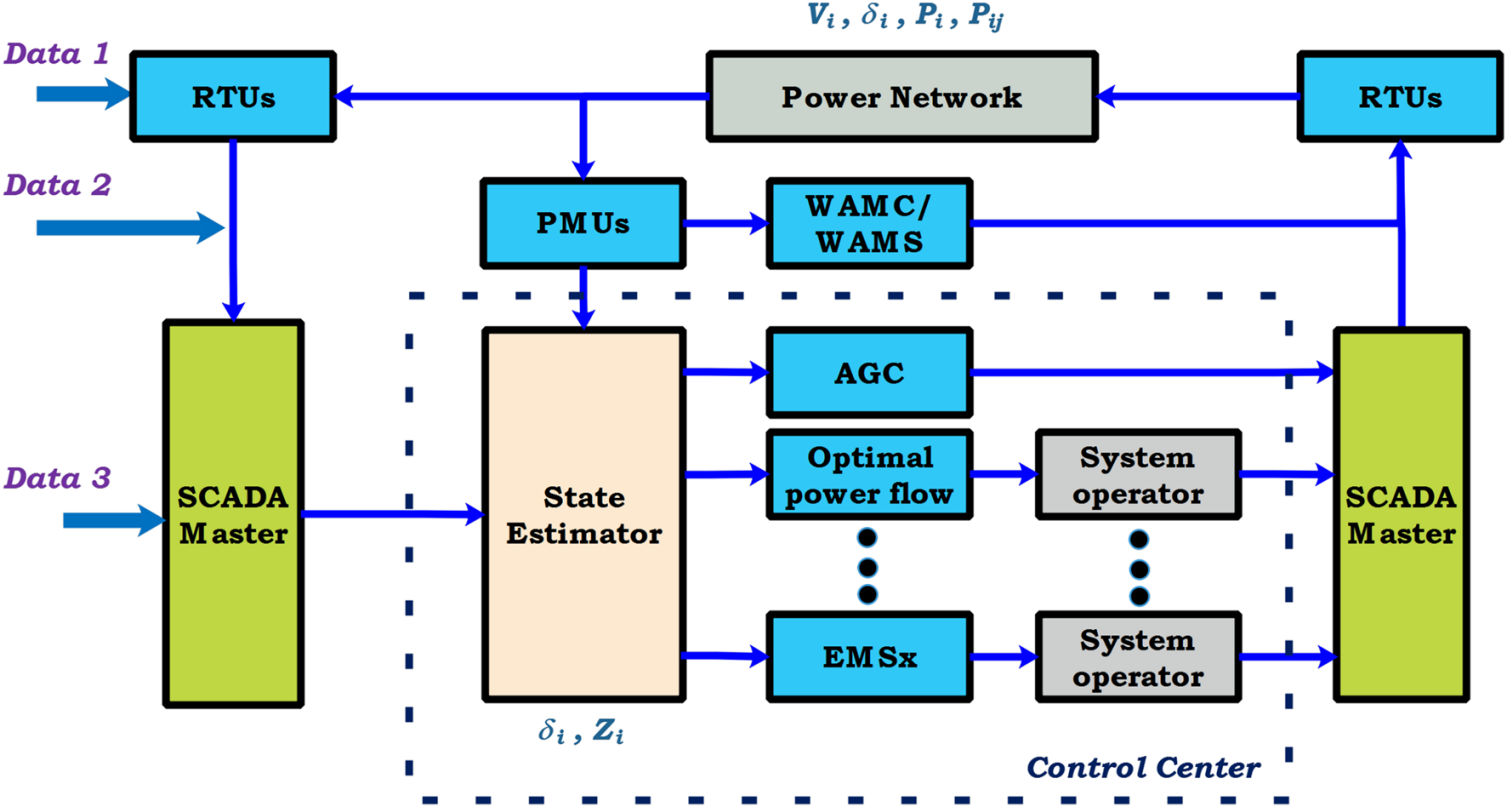
Data injection attack on power grid—a schematic view.

The attacker can access the output data of the sensor or operator and inject wrong information purposefully into the power grid control system to disrupt the state estimation process is termed as Data injection attack as shown in Fig. 10^85^.

**Figure 11:**
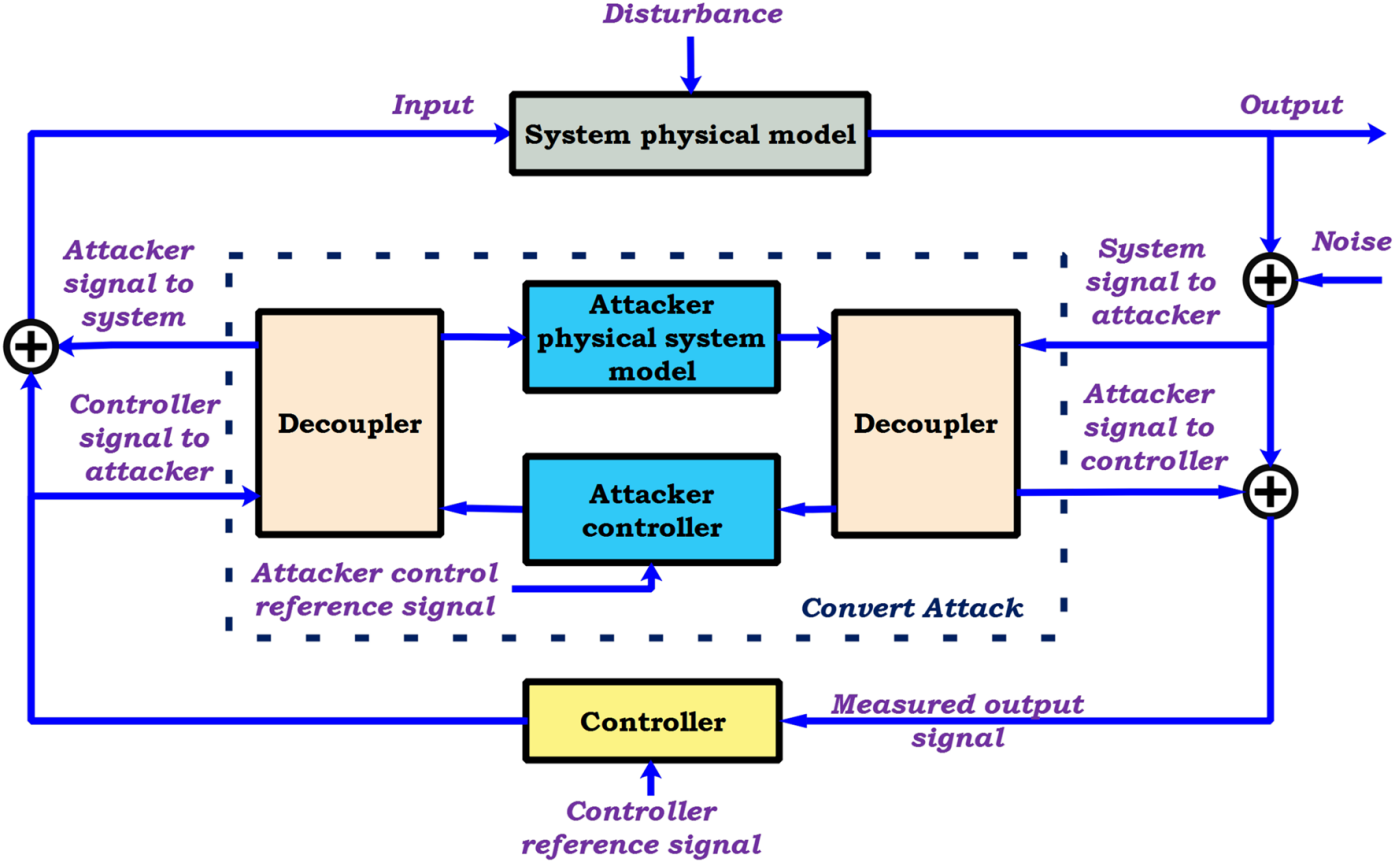
Stealth attack on power grid—a schematic view.

#### Invalid data injection attack

The attack aims to inject the wrong data dynamically and might destabilize the system. To encounter this issue, the Kalman filter and the linear quadratic gaussian (LQG) control scheme is used to detect a false dynamic data injection attack^86^.

#### Information recovery attack

The attacker records the communication network of field equipment under normal operation and sends it to the control network to disrupt the system at the time of the attack. One way to resolve this problem, mix a Gaussian random input with a mean of zero to the system input^87^.

#### Stealth attack in closed loop control system

With the knowledge of the physical model of the system, the attack output is reconstructed as hidden closed-loop information to eliminate the effect on the sensor output, as presented in Fig. 11^88^.

Therefore, IT-based security strategies alone cannot provide a defense-in-depth against cyber-attack in SGCS. Therefore, additional efforts are needed for identifying threats and risks to achieve optimal security in industrial control systems. The game theory approach is one of the most prominent methods used to estimate the security threats of cyber-physical systems^89^. In future investigations, it might be possible to use a cyber-physical networked microgrid management system for enhancing resiliency and responding to cyber adversarial events.

**Figure 12:**
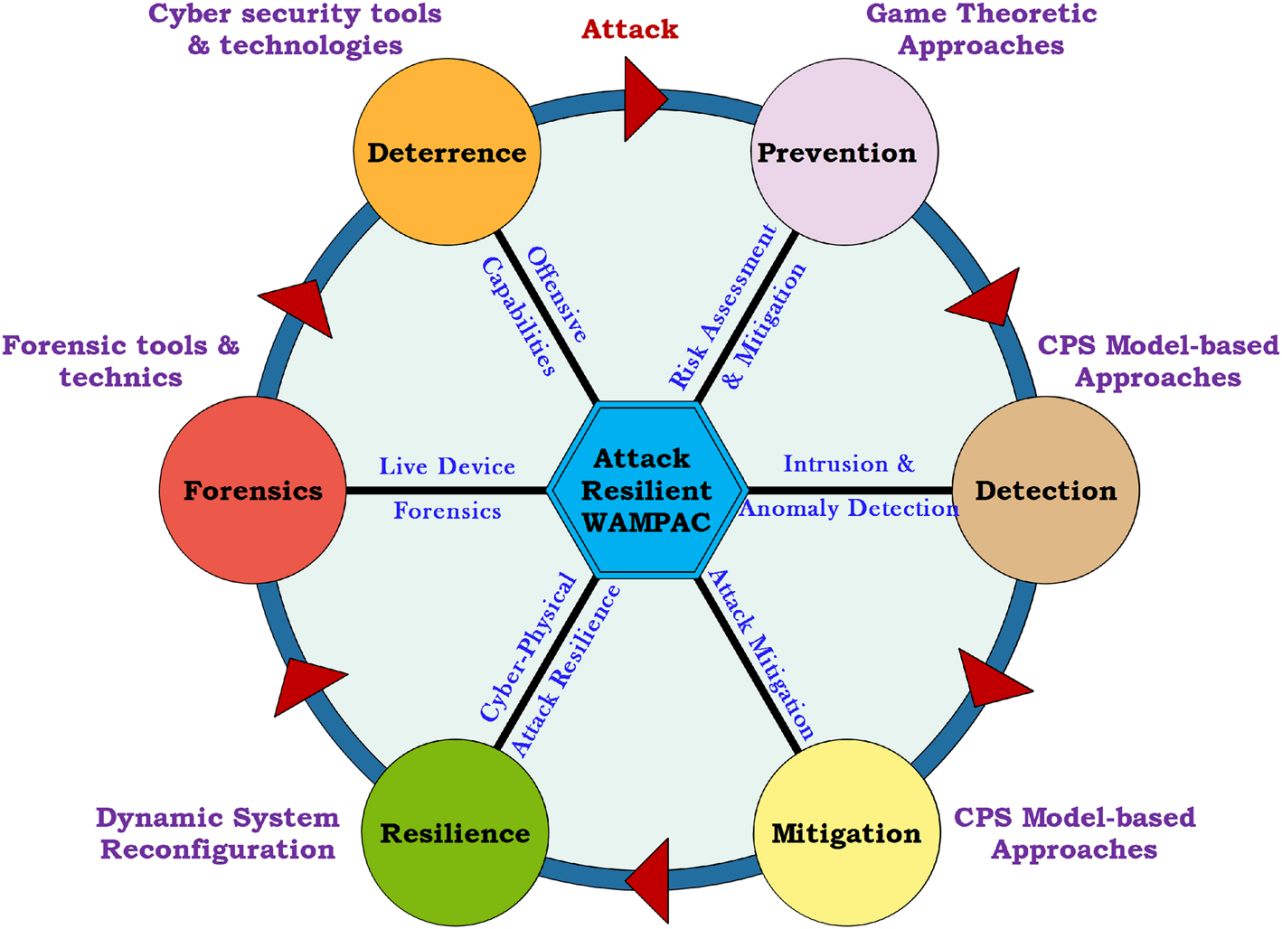
Attack-resilient WAMPAC for the power grid.

**Figure 13:**
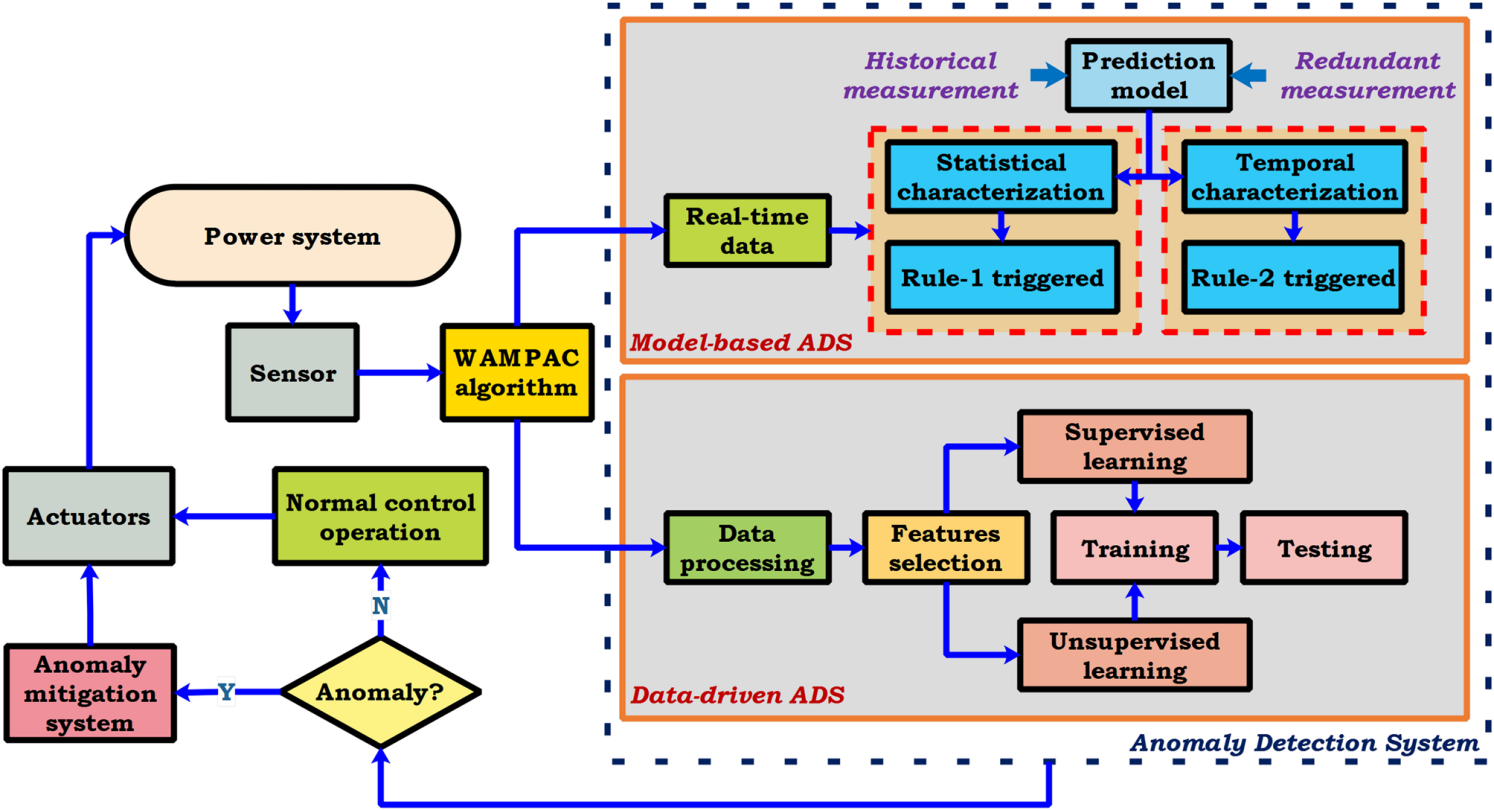
Attack-resilient system architecture for WAMPAC-EMS.

### Attack-resilient system in smart grid

A simple cyber-attack can disrupt the control network and may cause electrical blackouts. A reasonable approach to tackle this issue could be an attack-resilient grid infrastructure. The end-end security life cycle of attack resilient WAMPAC for the power grid is illustrated in Fig. 12. This approach can instantly detect stealthy cyber-attacks and provide an incident response to SGCS. Modern energy infrastructure can offer efficiency, reliability, stability, and sustainability operation to the electricity network. The SCADA systems are not suitable for real-time controls/time-critical applications due to skewed data, low scan rate, and extensive state estimation-time of magnitude and phasor measurements. Recently, modern grid control based on time-synchronized phasor data has developed to provide additional robustness. For providing real-time monitoring and control of the smart grid at the EMS layer (application layer) for WAMPAC applications, such as automatic generation control, state estimation, synchrophasor-based control strategy, remedial action scheme are recently developed to maintain the reliability and stability of power system. Among these strategies, synchrophasor technology is one of the most successful methods for data acquisition, transmitting, and processing. It consists of phasor measurement units (PMUs) in the field and phasor data concentrator (PDC). PMUs can provide voltage and current measurements with high resolution/speed at a specific location on the power grid. These measurements are time-synchronized phasor information every few milliseconds and record the data at 30 samples per second. PMU also detects faults early, allows for isolation of the operative system, and prevent the power outages. Thus, PMU determines the health of the system and increases reliability. The network of PMUs can provide real-time monitoring of power networks at the regional and national levels. PDC combines data from several measurement devices. However, due to the insecure data sharing equipment and unencrypted SCADA communication infrastructure, the existing vulnerabilities have been exposed to numerous cyber-attacks. Hence, an attack-resilient system (ARS) is essential to provide a defense-in-depth architecture against cyber-attack for the WAMPAC applications^90–96^.

$$\begin{aligned} Attack-Resilient\,System\,(ARS)\,=\,Anomaly\,Detection\,System\, (ADS) \,+ \,Attack \,Mitigation \,System \,(AMS) \end{aligned}$$

The conceptual attack-resilient system architecture is presented in Fig. 13 for WAMPAC-EMS applications. In ARS, the cyberattacks are detected in ADS based on anomalies in the observed data. Once the deviation is detected using either model-based ADS or data-driven ADS according to the type and nature of WAMPAC applications, AMS is activated depending on the situational awareness and possible corrective action against cyberattacks by adding advanced security layers in the application layers. Table. 4 shows the Control actions of ARS for different WAMPAC applications to prevent the propagation of cyberattacks in the smart grid network.

**Table 4:** Control actions of ARS for different WAMPAC applications.

WAMPAC	Control actions	Measurements and inputs	Data-driven ARS	Model-based ARS
Automatic generation control	Raise/lower generation	Frequency and tie-line power measurement	SCADA measurements, K-means clustering	Load forecasts
Remedial action scheme	Shed generation/load	Power/load measurement and lines status	Phasor measurements, decision tree (DT)	Multi-agent system (MAS)
State estimation	Generation redispatch and open/close breakers	Voltage and power,VAR or current-flow	Physics based features, dimension reduction technique	PMU measurements, load forecasts
Oscillations damping	FACTS, power system stabilizer (PSS), high voltage direct current system (HVDC)	Phasor measurements	Physics-based features, principal component analysis (PCA)	Redundant PMU measurements

Furthermore, the blockchain framework provides security and resiliency for the electrical power system. In the marker layer, trading transaction blockchain maintains all the transaction data of energy trading like tariff offering and bidding. At the ICT layer, aggregated data blockchain retains all the data in the ICT layer such as demand management, grid balancing, real-time monitoring, and smart metering. In the energy layer, sensors data blockchain keeps all the data of the energy grid such as generation, transmission, distribution, and consumption. In this blockchain framework, there is no direct link between the specific producer/consumer identity. Hence, the vulnerability against malicious attacks could be decreased. As a result, the electrical power network is more resilient^97^.

In terms of future work, research should focus on real-time cyber-attack identification, risk modeling, risk assessment analysis, RTDS testbed development with standard protocols, and energy management. Another possible area of future research would be the development of honeypots to capture malware in the power system. For more smart and secure smart meter infrastructure, a multi-agent-based distributed control system is further exemplified. The merits and outcomes of the various control strategies against cyber-attacks for smart grids are summarized in Table 5.

**Table 5:** Smart grid and microgrid resiliency.

Methodology	Control Method	Merits	Outcomes
IT based security strategies	• Cryptography, encryption, kalman filter, LQG controller, Gaussian random input and game theory	• Improve the quality, efficiency and reliability of smart grids	• Enhance cyber-Resilience in power system
		• Reduce the effect of attacks in power system	
Wide-area monitoring, protection, and control	• Attack-resilient system	• Prevent the propagation of cyber-attacks in the smart grid network	• Provide a real-time monitoring and grid condition-based automated responses to maintain the stability and reliability of power system

Overall, the essential components of a decentralized power system are distributed generation, demand response in transmission and distribution systems, and energy storage. Specifically, DESS can contribute to forming decentralized energy systems for promoting the generation, storing, and controlling energy locally and separated from the main grid. Besides, with the liberalization of energy markets, consumers are now option of choosing their electricity supplier. As a result, the number of electricity suppliers has increased significantly. The following are some of the more advanced and ready-to-use smart grid technologies: (1) distributed storage with power conditioning equipment and control is utilized for power conversion. (2) Smart inverters are used to identify operational and maintenance issues quickly. (3) AMI, including smart meters, can improve revenue collection, combat electricity theft, receive outage notifications, and schedule service and maintenance. (4) Demand response using AMI can shift peak loads, increase the system flexibility and make use of cheap time-of-use tariffs. (5) Transmission enhancement using superconductors, FACTS, and HVDC can improve network stability and automated recovery. These new technologies provide many benefits, including generation and delivery flexibility, better operational visibility, and more sophisticated planning and operation. However, methodologies for quantifying the value of these new technologies are still in a more immature state. More extensive and coordinated monitoring and communication could lead to greater system visibility of new technologies and resources across control areas.

## Discussion on future trends

Recent advances in the smart grid are discussed in this article, which includes communication enabled EV charging system, virtual Inertia control, smart inverters, big-data analytics, IoT based WSN and cyber-security for the power grid. Furthermore, some research progress has been made on synchrophasor-based wide-area monitoring and control^98–100^. Besides, the future studies in the smart grid should involve flash charging EV technology, reconfigurable distribution networks, and cyber resilience enhancement in power control systems. The future trends on the modernization of the electric power system can be summarized as follows:

### Smart EV charging infrastructure

In terms of directions for future research, further research should focus on the development of a hybrid input DC fast charger with a multi-input source with 510 kW single output for electric vehicle applications. Furthermore, it is essential to implement an AC level-2 charger with a 6.6 kW rating and dc fast charger with a 36 kW rating for each electric vehicle in the future^101,102^. It seems necessary to minimize the operating cost, maximize the reliability and controllability of the EV charging system. Therefore, the optimal battery charging method under the multi-car charging condition is one of the trends in future research. Another possible area of future research would be to investigate the incorporation of protection and communication features to the EV charger for enabling smart and secure EV charging system operation^103^.

The future trends on grid-supportive EV charger and charging infrastructure research and application at the LT level can be summarized as follows: (1) creating an IoT based communication infrastructure between EV users and charging stations, different entities in the system, and various EV charging stations with new concepts at a device and system level. (2) designing smart bi-directional EV chargers with/without photovoltaic capability. (3) developing advanced control strategies and net metering for V2G and G2V concepts. (4) formulating an Optimal charging algorithm to control EV charging/discharging. (5) implementing grid-supportive EV charging facilities on real-time feeders using efficient control strategies under various test conditions. Flash charging technology is an emerging trend in new-generation electric vehicles to save charging time at terminal feeding stations. It uses the on-board converter to provide a simple, reliable interface for efficient battery charging. This technology enables an emission-free public transport system with a minimized connection fee and energy cost through embedded peak shaving functionality^104–106^.

### Smart grid stability

Distributed control strategies are adopted to improve the small-signal stability of large power systems for maintaining a secure and reliable power grid. As far as the regulation and electricity market is concerned, different technical and scientific research solutions such as predictive (probabilistic) calculations, localization of switches, and the inclusion of demand-side control possibilities could serve as cost-effective options for adaptive load shedding schemes. Although the advanced technologies such as electric energy storage, synchrophasor, virtual inertia control, smart inverters, demand response, and electric vehicles, can ensure the stability of the power grid, the energy industry still requires advanced tools, standards, and guidelines for the secure, reliable and resilient operation of smart grid systems to ensure the grid stability, power quality, and cost-effectiveness on the entire value chain of the power network^107^.

Besides, the use of distributed demand-side-response (DDSR) comes with the need for Artificial Intelligence and Machine Learning solutions to regulate the power system in future research in this area. In the case of renewable power dips, grid operators require new tools to reduce power demand quickly. Meanwhile, smart distribution systems are adopted with advanced communication and control strategies for the prevention of blackouts. However, the optimal configuration of the distribution grid at hourly intervals is needed in the presence of renewable energy resources. Therefore, reconfigurable distribution networks with or without DER integration need to be developed, which should be capable of interfacing with various real-time simulators to facilitate system performance studies. This reconfiguration of electricity networks can bring benefits to both rural and developed networks during normal as well as emergencies^108^.

### Smart grid cyber resiliency

Synchrophasor based WAMPC system provides a new paradigm of resilient, efficient, and secure operation of the distribution system in the smart grid. For improving inter-area mode damping, impending system instability, and better situational awareness monitoring, PMUs need to be embedded with appropriate dynamic phasor estimation algorithms. In addition, suitable application tools should be developed for the successful implementation of WAMPC in a smart distribution system^109–111^.

Some research areas in cybersecurity should be the focus on (1) supply chain, threat, and third party authorized to access; (2) industry access to timely information on threats and vulnerabilities; (3) cloud/managed security service provider; (4) adequacy of security controls, and (5) internal network monitoring and detection. However, all cybersecurity solutions have been covered for slower operations (5–15 min) in power systems. These solutions cannot be accommodated in power electronic systems. Therefore, the resiliency capability of the power electronic system needs to be addressed in view of stability considerations for further studies^112–118^. Future research should focus on the feasibility study of recovery to the pre-attack point and relying on AI-based learning approaches for data authentication. Moreover, the impacts of cyber-attacks on communication links, master, and local controllers need to be investigated^119^.

## Conclusion

An overview of the extensive advanced technologies for the existing distribution network is presented to ensure that the power grid is smarter. The control concepts of energy storage need to be enhanced and some improved control methods are analyzed to mitigate the mismatch between renewable generation and load demand. Considering the energy storage technology in the DSO grid, this paper presents hysteresis and IMC-based voltage control. Although IMC significantly reduces voltage fluctuations of the affected feeders, further modifications are required to obtain the best results. As the grid instability problem is considered in damping control, BESS with appropriate grid-supportive inverters for active frequency damping control has been discussed in the current study. The control input measurements of damping control require a communication channel which increases the complexity of the system. Therefore, further research is required in decentralized control of BESS. In clean energy transportation, the impact of several types of energy storage systems on EV chargers is examined. Concerning densely populated areas, combat range anxiety, fast charging, and realize charge sustaining operation, this paper analyzes various charging technologies and their control methods for EVs. Consequently, EV provides the opportunity to facilitate the transition toward the electrification of the transportation sector. Further research should focus on supercapacitors and flash charging technology for providing faster charging to EV batteries. In addition, smart grid stability with advanced control concepts like virtual inertia, synchronverter, and data analytics in demand response applications are analyzed to improve frequency stability and mitigate the demand-supply gap of modern networks. Most existing studies in the field of energy management have only focused on developing models and sophisticated algorithms. Therefore, the ADMS approach has been discussed in this paper for better energy management and stability. Integrating ICT to advanced control systems with smart algorithm research is required for a better future of the smart grid. Furthermore, various cyber-attacks in SGCS have been analyzed in the present study. However, IT-based security solutions alone cannot provide a defense-in-depth against cyber-attack in SGCS. Therefore, the conceptual attack-resilient system with control actions for different WAMPAC has also been investigated to mitigate the vulnerability against malicious attacks. As a result, the electrical power network is more resilient. Future research should focus on artificial intelligence and machine learning-based real-time ARS for data authentication. Finally, future trends on the modernization of the electric power system are briefly discussed. Smart and secure grid-supportive EV Charging Infrastructure, reconfigurable distribution networks with or without DER integration, the resiliency capability of the power electronic system have become the mainstream trends. In addition, grid stability, cyber-security, power quality, power management, and cost-effectiveness on the entire value chain are still the main topics in the future research of smart power networks.

## References

[1] MajeedButt O, Zulqarnain M, MajeedButt T (2020). Recent advancement in smart grid technology: Future prospects in the electrical power network. Ain Shams Eng. J..

[2] Nadeem F, Hussain SMS, Tiwari PK, Goswami AK, Ustun TS (2019). Comparative review of energy storage systems, their roles, and impacts on future power systems. IEEE Access.

[3] Han S (2020). Selecting an effective ESS installation location from the perspective of reactive power. IEEE Access.

[4] Lamberti, F., Calderaro, V., Galdi, V. & Piccolo, A. Ancillary services provided by residential ESSS in LV networks: Assessing the opportunity costs. In *2017 IEEE Manchester PowerTech*, 1–6 (2017).

[5] Amamra S, Marco J (2019). Vehicle-to-grid aggregator to support power grid and reduce electric vehicle charging cost. IEEE Access.

[6] Morello R, Mukhopadhyay SC, Liu Z, Slomovitz D, Samantaray SR (2017). Advances on sensing technologies for smart cities and power grids: A review. IEEE Sens. J..

[7] Sun Q (2016). A comprehensive review of smart energy meters in intelligent energy networks. IEEE Internet Things J..

[8] Hasanuzzaman Shawon M, Muyeen SM, Ghosh A, Islam SM, Baptista MS (2019). Multi-agent systems in ICT enabled smart grid: A status update on technology framework and applications. IEEE Access.

[9] López G (2019). The role of power line communications in the smart grid revisited: Applications, challenges, and research initiatives. IEEE Access.

[10] Niyato D, Xiao L, Wang P (2011). Machine-to-machine communications for home energy management system in smart grid. IEEE Commun. Mag..

[11] Liu Z (2019). Transactive real-time electric vehicle charging management for commercial buildings with PV on-site generation. IEEE Trans. Smart Grid.

[12] Tran VT, Islam MR, Muttaqi KM, Sutanto D (2019). An efficient energy management approach for a solar-powered EV battery charging facility to support distribution grids. IEEE Trans. Ind. Appl..

[13] Gray S (2016). Cyber security in modern power systems defending the grid. IET Cyber Secur. Modern Power Syst..

[14] Ieee standard cybersecurity requirements for substation automation, protection, and control systems. *IEEE Std C37.240-2014* 1–38 (2015).

[15] Ieee draft guide for the interoperability of energy storage systems integrated with the electric power infrastructure. *IEEE P2030.2/D7.0, April 2014* 1–190 (2014).

[16] He H, Yan J (2016). Cyber-physical attacks and defences in the smart grid: A survey. IET Cyber-Phys. Syst. Theory Appl..

[17] Koltsaklis NE, Dagoumas AS (2018). State-of-the-art generation expansion planning: A review. Appl. Energy.

[18] Ibrahim MS, Dong W, Yang Q (2020). Machine learning driven smart electric power systems: Current trends and new perspectives. Appl. Energy.

[19] Wertani H, Ben Salem J, Lakhoua M (2020). Analysis and supervision of a smart grid system with a systemic tool. Electr. J..

[20] Li P (2020). Distributed adaptive robust voltage/var control with network partition in active distribution networks. IEEE Trans. Smart Grid.

[21] Ponočko J, Milanovič JV (2020). Multi-objective demand side management at distribution network level in support of transmission network operation. IEEE Trans. Power Syst..

[22] Martínez Sanz I (2019). Enhancing transmission and distribution system coordination and control in GB using power services from DERS. J. Eng..

[23] Edmunds C, Galloway S, Elders I, Bukhsh W, Telford R (2020). Design of a DSO-TSO balancing market coordination scheme for decentralised energy. IET Gener. Transm. Distrib..

[24] Yazdani S, Ferdowsi M, Shamsi P (2020). Internal model based smooth transition of a three-phase inverter between islanded and grid-connected modes. IEEE Trans. Energy Convers..

[25] Jazebi S, de León F, Nelson A (2020). Review of wildfire management techniques—Part I: Causes, prevention, detection, suppression, and data analytics. IEEE Trans. Power Deliv..

[26] Blaabjerg F, Yang Y, Yang D, Wang X (2017). Distributed power-generation systems and protection. Proc. IEEE.

[27] Zhu Y, Liu C, Sun K, Shi D, Wang Z (2019). Optimization of battery energy storage to improve power system oscillation damping. IEEE Trans. Sustain. Energy.

[28] Datta U, Kalam A, Shi J (2020). Battery energy storage system control for mitigating PV penetration impact on primary frequency control and state-of-charge recovery. IEEE Trans. Sustain. Energy.

[29] Ravichandran, A., Malysz, P., Sirouspour, S. & Emadi, A. The critical role of microgrids in transition to a smarter grid: A technical review. In *2013 IEEE Transportation Electrification Conference and Expo (ITEC)*, 1–7 (2013).

[30] Tulpule PJ, Marano V, Yurkovich S, Rizzoni G (2013). Economic and environmental impacts of a PV powered workplace parking garage charging station. Appl. Energy.

[31] Filho, J. C. R., Tiwari, A. & Dwivedi, C. Understanding the drivers of negative electricity price using decision tree. In *2017 Ninth Annual IEEE Green Technologies Conference (GreenTech)*, 151–156 (2017).

[32] Deng L, Hobbs BF, Renson P (2015). What is the cost of negative bidding by wind? A unit commitment analysis of cost and emissions. IEEE Trans. Power Syst..

[33] Dillon, J. & O’Malley, M. Data sensitivities for variable renewable energy curtailment estimation. In *2014 IEEE PES General Meeting | Conference Exposition*, 1–5 (2014).

[34] Baumert, R. & Epp, D. Hydrogen storage for fuel cell powered underwater vehicles. In *Proceedings of OCEANS ’93*, II/166–II/171 vol.2 (1993).

[35] Clairand J, Rodríguez-García J, Álvarez-Bel C (2018). Smart charging for electric vehicle aggregators considering users’ preferences. IEEE Access.

[36] Wang M, Mu Y, Shi Q, Jia H, Li F (2020). Electric vehicle aggregator modeling and control for frequency regulation considering progressive state recovery. IEEE Trans. Smart Grid.

[37] Clairand J, Rodríguez-García J, Álvarez-Bel C (2020). Assessment of technical and economic impacts of EV user behavior on EV aggregator smart charging. J. Modern Power Syst. Clean Energy.

[38] Mohamed AAS, Lashway CR, Mohammed O (2017). Modeling and feasibility analysis of quasi-dynamic WPT system for EV applications. IEEE Trans. Transp. Electrif..

[39] Mohamed AAS, Zhu L, Meintz A, Wood E (2020). Planning optimization for inductively charged on-demand automated electric shuttles project at Greenville, South Carolina. IEEE Trans. Ind. Appl..

[40] Mohsenzadeh, A., Pang, C., Pazouki, S. & Haghifam, M. Optimal siting and sizing of electric vehicle public charging stations considering smart distribution network reliability. In *2015 North American Power Symposium (NAPS)*, 1–6 (2015).

[41] Jia, L., Hu, Z., Song, Y. & Luo, Z. Optimal siting and sizing of electric vehicle charging stations. In *2012 IEEE International Electric Vehicle Conference*, 1–6 (2012).

[42] Liang Y, Guo C, Yang J, Ding Z (2020). Optimal planning of charging station based on discrete distribution of charging demand. IET Gener. Transm. Distrib..

[43] Sarker MR, Pandžić H, Ortega-Vazquez MA (2015). Optimal operation and services scheduling for an electric vehicle battery swapping station. IEEE Trans. Power Syst..

[44] Grigorescu, S. D., Ghita, O. M., Cepisca, C. & Vintea, A. S. Power quality monitoring systems for smart grid networks. In *2013 8th International SYmposium on Advanced Topics in Electrical Engineering (ATEE)*, 1–4 (2013).

[45] Chen C, Chen Y, Chin Y, Chen C (2018). Integrated power-quality monitoring mechanism for microgrid. IEEE Trans. Smart Grid.

[46] Li X, Wang S (2019). A review on energy management, operation control and application methods for grid battery energy storage systems. CSEE J. Power Energy Syst..

[47] IEEE recommended practice for interconnecting distributed resources with electric power systems distribution secondary networks. *IEEE Std 1547.6-2011* 1–38 (2011).

[48] Sharma D, Mishra S (2018). Power system frequency stabiliser for modern power systems. IET Gener. Transm. Distrib..

[49] SaeedUzZaman M (2020). Sensitivity and stability analysis of power system frequency response considering demand response and virtual inertia. IET Gener. Transm. Distrib..

[50] Obaid ZA, Cipcigan LM, Abrahim L, Muhssin MT (2019). Frequency control of future power systems: Reviewing and evaluating challenges and new control methods. J. Modern Power Syst. Clean Energy.

[51] Wang Y, Liu B, Duan S (2019). Modified virtual inertia control method of VSG strategy with improved transient response and power-supporting capability. IET Power Electron..

[52] Kerdphol T, Watanabe M, Hongesombut K, Mitani Y (2019). Self-adaptive virtual inertia control-based fuzzy logic to improve frequency stability of microgrid with high renewable penetration. IEEE Access.

[53] Rosso R, Engelken S, Liserre M (2019). Robust stability analysis of synchronverters operating in parallel. IEEE Trans. Power Electron..

[54] Vasudevan KR, Ramachandaramurthy VK, Babu TS, Pouryekta A (2020). Synchronverter: A comprehensive review of modifications, stability assessment, applications and future perspectives. IEEE Access.

[55] Roldán-Pérez J, Rodríguez-Cabero A, Prodanovic M (2019). Parallel current-controlled synchronverters for voltage and frequency regulation in weak grids. J. Eng..

[56] Kermani, H. R., Dahraie, M. V. & Najafi, H. R. Demand response strategy for frequency regulation in a microgrid without storage requirement. In *2016 24th Iranian Conference on Electrical Engineering (ICEE)*, 921–926 (2016).

[57] Wu Y-K, Tang K-T (2019). Frequency support by demand response—review and analysis. Energy Proced..

[58] Hafeez G (2020). An innovative optimization strategy for efficient energy management with day-ahead demand response signal and energy consumption forecasting in smart grid using artificial neural network. IEEE Access.

[59] Hadi AA, Silva CAS, Hossain E, Challoo R (2020). Algorithm for demand response to maximize the penetration of renewable energy. IEEE Access.

[60] Jamil M, Mittal S (2020). Hourly load shifting approach for demand side management in smart grid using grasshopper optimisation algorithm. IET Gener. Transm. Distrib..

[61] Khan ZA, Jayaweera D (2020). Smart meter data based load forecasting and demand side management in distribution networks with embedded PV systems. IEEE Access.

[62] Pratt, A. *et al.* A test bed to evaluate advanced distribution management systems for modern power systems. In *IEEE EUROCON 2019—18th International Conference on Smart Technologies*, 1–6 (2019).

[63] Boardman E (2020). Advanced applications in an advanced distribution management system: Essentials for implementation and integration. IEEE Power Energy Mag..

[64] Dubey A, Bose A, Liu M, Ochoa LN (2020). Paving the way for advanced distribution management systems applications: Making the most of models and data. IEEE Power Energy Mag..

[65] Wang Y, Chen Q, Hong T, Kang C (2019). Review of smart meter data analytics: Applications, methodologies, and challenges. IEEE Trans. Smart Grid.

[66] Bhattarai BP (2019). Big data analytics in smart grids: State-of-the-art, challenges, opportunities, and future directions. IET Smart Grid.

[67] Huang AQ (2017). Power semiconductor devices for smart grid and renewable energy systems. Proc. IEEE.

[68] Ding X, Zhou Y, Cheng J (2019). A review of gallium nitride power device and its applications in motor drive. CES Trans. Electr. Mach. Syst..

[69] Khaligh A, D’Antonio M (2019). Global trends in high-power on-board chargers for electric vehicles. IEEE Trans. Vehic. Technol..

[70] Zeadally S, Adi E, Baig Z, Khan IA (2020). Harnessing artificial intelligence capabilities to improve cybersecurity. IEEE Access.

[71] Buque, C. & Chowdhury, S. Distributed generation and microgrids for improving electrical grid resilience: Review of the mozambican scenario. In *2016 IEEE Power and Energy Society General Meeting (PESGM)*, 1–5 (2016).

[72] Wang Z, Chen B, Wang J, Chen C (2016). Networked microgrids for self-healing power systems. IEEE Trans. Smart Grid.

[73] Wang Z, Chen B, Wang J, Begovic MM, Chen C (2015). Coordinated energy management of networked microgrids in distribution systems. IEEE Trans. Smart Grid.

[74] Wang Z, Chen B, Wang J, Kim J (2016). Decentralized energy management system for networked microgrids in grid-connected and islanded modes. IEEE Trans. Smart Grid.

[75] Zhang Q, Dehghanpour K, Wang Z, Huang Q (2020). A learning-based power management method for networked microgrids under incomplete information. IEEE Trans. Smart Grid.

[76] Wang Z, Wang J (2015). Self-healing resilient distribution systems based on sectionalization into microgrids. IEEE Trans. Power Syst..

[77] Zhao, Z. & Chen, G. An overview of cyber security for smart grid. In *2018 IEEE 27th International Symposium on Industrial Electronics (ISIE)*, 1127–1131 (2018).

[78] Igure VM, Laughter SA, Williams RD (2006). Security issues in SCADA networks. Comput. Secur..

[79] Wang B, Dabbaghjamanesh M, Kavousi-Fard A, Mehraeen S (2019). Cybersecurity enhancement of power trading within the networked microgrids based on blockchain and directed acyclic graph approach. IEEE Trans. Ind. Appl..

[80] Chen Y, Hong J, Liu C (2018). Modeling of intrusion and defense for assessment of cyber security at power substations. IEEE Trans. Smart Grid.

[81] Puthal D, Wu X, Surya N, Ranjan R, Chen J (2019). Seen: A selective encryption method to ensure confidentiality for big sensing data streams. IEEE Trans. Big Data.

[82] Xu, Y., Yang, Y., Li, T., Ju, J. & Wang, Q. Review on cyber vulnerabilities of communication protocols in industrial control systems. In *2017 IEEE Conference on Energy Internet and Energy System Integration (EI2)*, 1–6 (2017).

[83] Arghandeh R, Alexandr ML, Mili L (2016). On the definition of cyber-physical resilience in power systems. Renew. Sustain. Energy Rev..

[84] Yang Q (2014). On false data-injection attacks against power system state estimation: Modeling and countermeasures. IEEE Trans. Parallel Distrib. Syst..

[85] Monticelli A (2012). State Estimation in Electric Power Systems: A Generalized Approach.

[86] Manandhar K, Cao X, Hu F, Liu Y (2014). Detection of faults and attacks including false data injection attack in smart grid using kalman filter. IEEE Trans. Control Netw. Syst..

[87] Mehrdad S, Mousavian S, Madraki G, Dvorkin Y (2018). Cyber-physical resilience of electrical power systems against malicious attacks: A review. Curr. Sustain. Renew. Energy Rep..

[88] Dán, G. & Sandberg, H. Stealth attacks and protection schemes for state estimators in power systems. In *2010 First IEEE International Conference on Smart Grid Communications*, 214–219 (2010).

[89] Amin S, Schwartz GA, Hussain A (2013). In quest of benchmarking security risks to cyber-physical systems. IEEE Netw..

[90] Ashok A, Govindarasu M, Wang J (2017). Cyber-physical attack-resilient wide-area monitoring, protection, and control for the power grid. Proc. IEEE.

[91] Sridhar S, Govindarasu M (2014). Model-based attack detection and mitigation for automatic generation control. IEEE Trans. Smart Grid.

[92] Wang, P., Govindarasu, M., Ashok, A., Sridhar, S. & McKinnon, D. Data-driven anomaly detection for power system generation control. In *2017 IEEE International Conference on Data Mining Workshops (ICDMW)*, 1082–1089 (2017).

[93] Singh, V. K., Ozen, A. & Govindarasu, M. A hierarchical multi-agent based anomaly detection for wide-area protection in smart grid. In *2018 Resilience Week (RWS)*, 63–69 (2018).

[94] Singh, V. K. & Govindarasu, M. Decision tree based anomaly detection for remedial action scheme in smart grid using pmu data. In *2018 IEEE Power Energy Society General Meeting (PESGM)*, 1–5 (2018).

[95] Ashok A, Govindarasu M, Ajjarapu V (2018). Online detection of stealthy false data injection attacks in power system state estimation. IEEE Trans. Smart Grid.

[96] Mahapatra K, Ashour M, Chaudhuri NR, Lagoa CM (2020). Malicious corruption resilience in PMU data and wide-area damping control. IEEE Trans. Smart Grid.

[97] Liang G, Weller SR, Luo F, Zhao J, Dong ZY (2019). Distributed blockchain-based data protection framework for modern power systems against cyber attacks. IEEE Trans. Smart Grid.

[98] Almas MS, Vanfretti L, Singh RS, Jonsdottir GM (2018). Vulnerability of synchrophasor-based WAMPAC applications’ to time synchronization spoofing. IEEE Trans. Smart Grid.

[99] Cui H, Li F, Tomsovic K (2020). Cyber-physical system testbed for power system monitoring and wide-area control verification. IET Energy Syst. Integr..

[100] Bagdadee AH, Hoque MZ, Zhang L (2020). Iot based wireless sensor network for power quality control in smart grid. Proced. Comput. Sci..

[101] Hammami, M., Viatkin, A., Ricco, M. & Grandi, G. A dc/dc fast charger for electric vehicles with minimum input/output ripple based on multiphase interleaved converters. In *2019 International Conference on Clean Electrical Power (ICCEP)*, 187–192 (2019).

[102] Dao ND, Lee D, Phan QD (2020). High-efficiency sic-based isolated three-port DC/DC converters for hybrid charging stations. IEEE Trans. Power Electron..

[103] Antoun J, Kabir ME, Moussa B, Atallah R, Assi C (2020). A detailed security assessment of the EV charging ecosystem. IEEE Netw..

[104] Kulkarni, B., Patil, D. & Suryavanshi, R. G. Iot based PV assisted EV charging station for confronting duck curve. In *2018 International Conference on Computational Techniques, Electronics and Mechanical Systems (CTEMS)*, 36–39 (2018).

[105] Sudheer, K., Kumar, K. H., Puneethkumar, N. & Reddy, K. V. Iot based intelligent smart controller for electric vehicles. In *2020 6th International Conference on Advanced Computing and Communication Systems (ICACCS)*, 539–544 (2020).

[106] Alessandrini, A. *et al.* A flash charge system for urban transport. In *2019 IEEE International Conference on Environment and Electrical Engineering and 2019 IEEE Industrial and Commercial Power Systems Europe (EEEIC/I CPS Europe)*, 1–6 (2019).

[107] Radoglou-Grammatikis PI, Sarigiannidis PG (2019). Securing the smart grid: A comprehensive compilation of intrusion detection and prevention systems. IEEE Access.

[108] Akrami A, Doostizadeh M, Aminifar F (2020). Optimal reconfiguration of distribution network using $$\mu $$ PMU measurements: A data-driven stochastic robust optimization. IEEE Trans. Smart Grid.

[109] Xu S, Liu H, Bi T, Martin KE (2020). A high-accuracy phasor estimation algorithm for PMU calibration and its hardware implementation. IEEE Trans. Smart Grid.

[110] Biswal, M., Misra, S. & Tayeen, A. S. Black box attack on machine learning assisted wide area monitoring and protection systems. In *2020 IEEE Power Energy Society Innovative Smart Grid Technologies Conference (ISGT)*, 1–5 (2020).

[111] Pei C, Xiao Y, Liang W, Han X (2020). PMU placement protection against coordinated false data injection attacks in smart grid. IEEE Trans. Ind. Appl..

[112] Sahoo S, Mishra S, Peng JC, Dragičevič T (2019). A stealth cyber-attack detection strategy for dc microgrids. IEEE Trans. Power Electron..

[113] Sahoo S, Peng JC, Devakumar A, Mishra S, Dragičević T (2020). On detection of false data in cooperative dc microgrids—a discordant element approach. IEEE Trans. Ind. Electron..

[114] Sahoo S, Peng JC, Mishra S, Dragičević T (2020). Distributed screening of hijacking attacks in dc microgrids. IEEE Trans. Power Electron..

[115] Sahoo S, Dragičević T, Blaabjerg F (2020). Resilient operation of heterogeneous sources in cooperative dc microgrids. IEEE Trans. Power Electron..

[116] Sahoo S, Peng JC (2020). A localized event-driven resilient mechanism for cooperative microgrid against data integrity attacks. IEEE Trans. Cybern..

[117] Sahoo S, Dragičević T, Blaabjerg F (2020). An event-driven resilient control strategy for dc microgrids. IEEE Trans. Power Electron..

[118] Sahoo S, Dragicevic T, Blaabjerg F (2019). Cyber security in control of grid-tied power electronic converters-challenges and vulnerabilities. IEEE J. Emerg. Sel. Top. Power Electron..

[119] Gupta R (2020). Smart contract privacy protection using AI in cyber-physical systems: Tools, techniques and challenges. IEEE Access.

